# Guided Internet-Based Cognitive Behavior Therapy for Women With Bulimia Nervosa: A Randomized Clinical Trial

**DOI:** 10.1001/jamanetworkopen.2025.25165

**Published:** 2025-08-05

**Authors:** Sayo Hamatani, Kazuki Matsumoto, Gerhard Andersson, Yasuhiro Sato, Shin Fukudo, Yusuke Sudo, Yoshiyuki Hirano, Keiko Ino, Tomoaki Ishibashi, Yukiko Tomioka, Hidehiro Umehara, Shusuke Numata, Masayuki Nakamura, Ryoko Otani, Ryoichi Sakuta, Atsushi Sekiguchi, Hirotaka Kosaka, Yoshifumi Mizuno, and the HOPE Project Consortium, Rio Kamashita, Tokiko Yoshida, Kanae Matsuura, Shinji Tomari, Misako Funaba, Natsuki Sasaki, Haruka Sako, Shoko Shimada, Takeshi Inoue

**Affiliations:** 1 Research Center for Child Mental Development, University of Fukui, Fukui, Japan; 2 Division of Developmental Higher Brain Functions, United Graduate School of Child Development, University of Fukui, Fukui, Japan; 3 Department of Child and Adolescent Psychological Medicine, University of Fukui Hospital, Fukui, Japan; 4 Division of Clinical Psychology, Kagoshima University Hospital, Research and Education Assembly Medical and Dental Sciences Area, Kagoshima University, Kagoshima, Japan; 5 Department of Behavioural Sciences and Learning, Linköping University, Linköping, Sweden; 6 Department of Biomedical and Clinical Science, Linköping University, Linköping, Sweden; 7 Department of Clinical Neuroscience, Karolinska Institute, Stockholm, Sweden; 8 Department of Psychosomatic Medicine, Tohoku University Hospital, Sendai, Japan; 9 Research Center for Child Mental Development, Chiba University, Chiba, Japan; 10 Division of Medical Treatment and Rehabilitation, Center for Forensic Mental Health, Chiba University, Chiba, Japan; 11 United Graduate School of Child Development, Osaka University, Kanazawa University, Hamamatsu University, Chiba University, and University of Fukui, Osaka, Japan; 12 Department of Behavioral Medicine, National Institute of Mental Health, National Center of Neurology and Psychiatry, Tokyo, Japan; 13 Department of Neuropsychiatry, University of Fukui, Fukui, Japan; 14 Department of Psychiatry, Graduate School of Biomedical Sciences, Tokushima University, Tokushima, Japan; 15 Department of Psychiatry, Graduate School of Medical and Dental Sciences, Kagoshima University, Kagoshima, Japan; 16 Child Development and Psychosomatic Medicine Center, Dokkyo Medical University Saitama Medical Center, Saitama, Japan; 17 Medical Affairs Division, University of Tokushima Hospital, Tokushima, Japan; 18 Student Counseling Center, Meiji Gakuin University, Tokyo, Japan

## Abstract

**Importance:**

Despite the rising prevalence of bulimia nervosa and the associated risks of chronicity and severe physical and psychological morbidity, access to effective treatment remains poor. The effectiveness and acceptability of internet-based cognitive behavior therapy (ICBT) for women with bulimia nervosa in clinical settings in East Asia remain unclear.

**Objective:**

To determine the effectiveness and acceptability of a guided ICBT program to treat women with bulimia nervosa in Japan.

**Design, Setting, and Participants:**

This randomized clinical trial was conducted at 7 university hospitals in Japan between August 2022 and October 2024. This study enrolled female participants aged 13 to 65 years whose symptoms met the *Diagnostic and Statistical Manual of Mental Disorders* (Fifth Edition) criteria for bulimia nervosa, had a body mass index (BMI) of 17.5 or greater, had internet access, and had no history of practicing CBT-related techniques within the past 2 years.

**Interventions:**

Both the control and intervention groups received usual care. The intervention consisted of ICBT with additional guidance from a therapist. The therapy program was tailored to Japanese culture and grounded in a specific cognitive behavior model, and it was performed over a 12-week period.

**Main Outcomes and Measures:**

Severity of bulimia nervosa, measured by the weekly combined frequency of episodes involving binge eating and compensatory behaviors, was assessed by a blinded, independent rating team at baseline and at the 12-week intervention end point. Intention-to-treat analyses were conducted using a linear mixed model with effect sizes calculated using Cohen *d*.

**Results:**

A total of 61 women met the eligibility criteria and were randomized to the intervention group (n = 31) or the control group (n = 30). Participants were predominantly young (mean [SD] age, 27.8 [9.0] years), had normal weight (mean [SD] BMI, 21.1 [3.6]), and had a mean (SD) duration of illness of 9.3 (8.8) years; half (31 [50.8%]) were employed. Intent-to-treat analysis revealed that guided ICBT significantly reduced the weekly combined frequency of episodes involving binge eating and compensatory behaviors (by an adjusted mean difference of 9.84 episodes [95% CI, 2.49-17.18 episodes], *P* = .01; Cohen *d* = 0.73 [95% CI, 0.21-1.26]). Sensitivity analyses supported these findings.

**Conclusions and Relevance:**

In this randomized clinical trial, the intervention group experienced a significant decrease in bulimia symptoms compared with the control group, supporting the effectiveness and acceptability of the therapist-guided ICBT program. These findings suggest that integration of therapist-guided ICBT in usual care has the potential to improve accessibility to efficacious treatment options for women with bulimia nervosa.

**Trial Registration:**

UMIN Clinical Trials Registry Identifier: UMIN00048732

## Key Points

**Question:**

Is guided internet-based cognitive behavior therapy (ICBT) effective in the treatment of women with bulimia nervosa in an outpatient psychiatric setting?

**Findings:**

In this randomized clinical trial of 61 women with bulimia nervosa, women who received ICBT plus usual care had a significant decrease in bulimia symptoms compared with women who received usual care only.

**Meaning:**

These findings support guided ICBT as a promising intervention for women with bulimia nervosa receiving treatment in psychiatric settings.

## Teaser

This randomized clinical trial conducted in Japan evaluates the effectiveness of a guided internet-based cognitive behavior therapy program to treat women with bulimia nervosa.

## Introduction

Bulimia nervosa (BN) is a mental health disorder characterized by binge eating, a distorted body image, and excessive preoccupation with low weight and a thin body shape.^1^ Evidence indicates that up to 3% of females suffer from BN during their lifetime, implicating an approximate 5-fold increase in mortality risk.^2^ Despite understanding the potential health risks, individuals with eating disorders often restrict their meals or engage in excessive exercise in pursuit of an ideal body shape and weight.^3,4^ Chronic abnormal eating behaviors and inappropriate compensatory behaviors can lead to severe physical and psychological damage.^5,6,7,8,9^ Given the poor prognosis of BN, early access to evidence-based treatments is crucial.

Research suggests that face-to-face individual cognitive behavior therapy (CBT) is effective in treating BN.^10,11^ The cognitive behavior model for eating disorders works by modifying patients’ dysfunctional beliefs about dieting and body shape and weight.^12^ Studies have suggested that the cognitive behavior model may also be effective when delivered via internet-based CBT (ICBT) with a therapist guide,^13^ helping reduce the frequency of binge eating and compensatory behaviors in individuals with BN.^14^ In addition to this evidence of efficacy, internet-based treatment is an attractive approach given the current poor access to standard treatment for BN.

However, evidence from randomized clinical trials (RCTs) for guided ICBT for BN has been limited to Germany, Sweden, the Netherlands, and Australia.^14,15,16,17^ To our knowledge, the efficacy of guided ICBT for BN has not yet been examined in Eastern cultures. Furthermore, only 1 RCT has investigated the efficacy of guided ICBT as an add-on to usual care (UC).^14^ Investigating the effectiveness of guided ICBT in clinical settings is crucial for bridging the gap between research and clinical practice. Further, 3 of the aforementioned RCTs had a few limitations, including insufficient sample sizes^16^ and potential confounding factors due to the inclusion of participants with binge eating disorders.^15,17^

Against this background, the current study aimed to examine the effectiveness of guided ICBT in treating BN in a clinical setting in Japan. We hypothesized (1) that integrating the guided ICBT program in the UC regime would be more effective than providing UC alone and (2) that a guided ICBT program for BN, tailored to the cultural context of Japan, would be acceptable to Japanese women with BN.

## Methods

The trial protocol for this RCT was reviewed and approved by the Research Ethics Committee of the University of Fukui (Supplement 1). Written informed consent was obtained from all participants. Before the study commenced, the clinical trial protocol was registered at the UMIN Clinical Trials Registry (UMIN00048732). We report the findings of this clinical trial in accordance with the CONSORT-Outcomes 2022 Extension^18^ of the Consolidated Standards of Reporting Trials (CONSORT) reporting guideline.

### Setting and Participants

This prospective multicenter RCT was designed in Japan, with participants recruited between August 2022 and July 2024 and follow-up completed in October 2024. To bridge the gap between existing research evidence and clinical practice, outpatients undergoing treatment at psychiatric clinics in Japan were recruited. Screening for eligibility and exclusion criteria was conducted using the Mini International Neuropsychiatric Interview.^19,20^ The eligibility criteria were as follows: female sex, age 13 to 65 years, diagnosis of BN according to *Diagnostic and Statistical Manual of Mental Disorders* (Fifth Edition) criteria,^1^ body mass index (BMI; calculated as weight in kilograms divided by height in meters squared) of 17.5 or greater, internet access (via tablet, personal computer, or smartphone), and no history of practicing CBT-related techniques within the past 2 years. The exclusion criteria were as follows: diagnosis of severe mental disorders (eg, psychosis), imminent risk of suicide, antisocial behavior, severe invasive physical illness, or refusal to be exposed to a feared object (eMethods in Supplement 2). Regarding ongoing treatments, the use of medications (eg, selective serotonin reuptake inhibitors) and psychosocial support (eg, counseling) were permitted during the study. However, participants were requested to refrain from making changes to these treatments during the study period. The eligibility assessment process is described in the eMethods in Supplement 2.

### Sample Size

The sample size was calculated using the G*Power free software package, version 3.1 (Heinrich-Heine-Universität Düsseldorf). An effect size of Cohen *d* = 0.90 from a previous study was used as a reference.^21^ The significance level was set at *P* < .05 for 2-sided tests. Power was set to 0.8. The required sample size (assigned number of participants) was determined to be 60, accounting for a 30% noncompletion rate based on a previous study.^22^

### Study Design and Procedures

The protocol for this prospective multicenter RCT was previously outlined in a published protocol article.^23^ The original unpublished protocol is provided in Supplement 1, and updates of the original protocol are provided in the eMethods in Supplement 2. Seven university hospitals in Japan collaborated to recruit outpatients from local psychiatric clinics. A dedicated website was created to publicize the study, allowing potential participants to enroll online. Furthermore, participants were recruited through online advertisements posted on various social media platforms.

### Intervention Arms

A culturally adapted ICBT program for Japanese women with BN served as the intervention.^24^ On a weekly basis, a CBT module was released as scheduled, with automated emails encouraging participants to engage in the program. The ICBT platform included an asynchronous messaging feature for therapist-participant communication,^25^ and the therapist (S.H.) responded within 24 hours. The specific techniques implemented in the ICBT program are detailed in Supplement 1, and the user interface of the BN ICBT program is presented in the eFigure in Supplement 2.

### Outcomes

The primary outcome—the weekly combined frequency of binge eating and compensatory behavior episodes—was assessed at baseline and 12 weeks post intervention. An independent assessor team from the University of Fukui measured the primary outcome via telephone calls or in person with the participants (eMethods in Supplement 2).

Secondary outcomes included (1) the weekly frequency of binge eating and compensatory behavior episodes measured separately and (2) scores on the following scales: the Eating Disorder Examination Questionnaire (EDE-Q),^26,27^ the Patient Health Questionnaire-9 (PHQ-9),^28,29^ the Generalized Anxiety Disorder-7 scale (GAD-7),^29,30^ the Brunnsviken Brief Quality of Life Scale (BBQ),^31,32^ the EuroQol 5 Dimensions 5 Levels (EQ-5D-5L),^33,34^ the Client Satisfaction Questionnaire (CSQ),^35,36^ and the Working Alliance Inventory—Short Form (WAI-SF).^37,38^ These self-rating scales were administered at baseline and 12 weeks post intervention within the ICBT system.

The EDE-Q is a 28-item self-report measure of eating disorder symptoms over the past 4 weeks, comprising 4 subscales: restraint (mean [SD], 0.99 [1.20]), eating concern (mean [SD], 0.61 [0.82]), shape concern (mean [SD], 2.54 [1.48]), and weight concern (mean [SD], 2.20 [1.38]). The EDE-Q global score (mean [SD], 1.71 [1.07] in Japanese female university students) is the average of these 4 subscales.^27^ The PHQ-9 and the GAD-7 are 9- and 7-item measures using 4-point Likert scales, yielding total scores of 0 to 27 and 0 to 21, respectively.^28,30^ The BBQ assesses subjective quality of life (QOL) via 12 items across 6 domains (leisure, view on life, creativity, learning, friends, and view on self), with a total score of 0 to 96.^31^ The EQ-5D-5L assesses QOL across 5 dimensions (mobility, self-care, usual activities, pain/discomfort, and anxiety/depression) using a 5-point scale, scored from 1 (no problems) to 5 (extreme problems). A quality of adjusted life-years index from 0 (death) to 1 (full health) can be derived.^33^ The CSQ-8 yields a total score of 8 to 32 by summing ratings across 8 items on service quality, met needs, and satisfaction.^36^ The WAI-SF is a 12-item measure of the therapeutic alliance (bond and agreement on goals/tasks), scored from 12 to 84.^37,38^

In this study, we adopted 2 criteria for defining BN remission. Cutoff scores of 2.34 and 2.80, derived from the EDE-Q global score, were adopted as thresholds between a healthy level and a clinically significant state,^39,40^ indicating 2 remission thresholds.

The secondary outcome measures relating to clinical symptoms were assessed at baseline and post intervention. The measures of satisfaction and therapeutic alliance were assessed only at the end of the 12-week intervention. Detailed information on these measures is provided in Supplement 1.

### Randomization and Blinding

An independent data management team at the University of Fukui randomized all participants to either the intervention group or the control group using the UMIN Medical Research Support (Case Registration and Allocation) System Cloud^41^ and forced balanced randomization. Owing to complete randomization, the last 5 cases were consecutively assigned to the intervention group, as the control group reached the target sample size first. To ensure concealment, neither the participants nor clinicians were aware of the randomization sequence. The participants were immediately informed of their group assignment. Participants allocated to the control group were informed that they would have access to the intervention after a waiting period. Furthermore, an independent evaluation team at the University of Fukui was blinded to the group assignment of participants. The success of blinding was evaluated using the methods of both Bang et al^42^ and James et al.^43^ The eMethods in Supplement 2 describes the procedures used to assess the success of blinding.

### Statistical Analysis

To assess the baseline differences between the 2 groups, we used independent-sample *t* tests, χ^2^ tests, and Fisher exact tests, as appropriate. Following the intention-to-treat (ITT) principle,^44^ we applied a linear mixed model (LMM) with sites and participants as random effects and time (pre vs post), treatment (intervention vs control), and time × treatment interaction as fixed effects.^45^ This model was adjusted for baseline scores and covariates including age, duration of illness, years of education, and comorbidities. The treatment effect was derived from the treatment × time interaction and standardized using Cohen *d* to calculate the effect size.^46^ The primary outcome was the least-squares mean difference estimate, with a 95% CI. For the dichotomous outcome for BN remission, we used the χ^2^ test to confirm the significance of the odds ratio (OR).

In addition to the ITT analysis, a per-protocol set (PPS) analysis was conducted to evaluate the robustness of the findings. In the PPS analysis, the participants who adhered to the treatments as planned were included. In the intervention group, treatment adherence was defined as accessing 80% or more of the ICBT program content and completing 40% or more of the worksheets, typically equivalent to 5 modules. In the control group, treatment adherence was defined as continuing UC during the RCT.

Two sensitivity analyses were performed for all outcomes. The initial sensitivity analysis employed an analysis of covariance (ANCOVA) with baseline scores, age, institution, duration of illness, comorbidities, and educational history as covariates to assess the robustness of the findings.^47^ For the secondary sensitivity analysis, the LMM was applied to the dataset after the imputation of missing values to explore the impact of missing data on the results. Multiple imputation with 20 simulations was performed to address missing data. We conducted observed power analysis to assess the validity of our primary results.

All statistical analyses were conducted using IBM SPSS Statistics, version 29 (IBM SPSS). R, version 4.4.0 (R Project for Statistical Computing),^48^ was used for graphical representations (plotly package)^49^ and for evaluating blinding integrity using the BI package.^50^ Two-sided *P* values were considered statistically significant at *P* < .05.

## Results

### Study Participants

**Figure 1.**
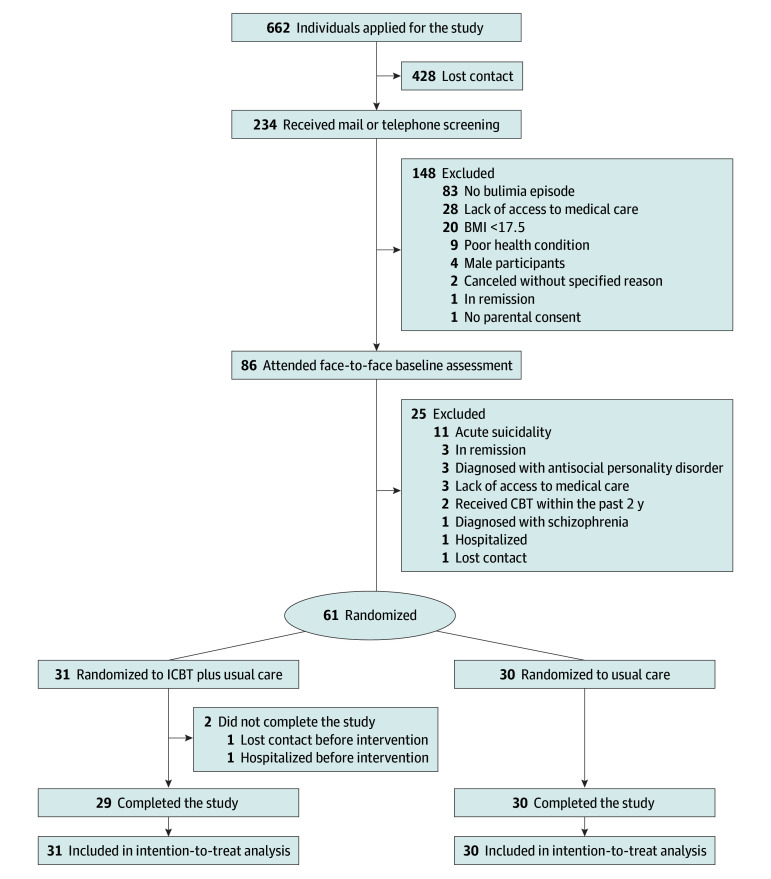
Participant Flow Diagram BMI indicates body mass index (calculated as weight in kilograms divided by height in meters squared); CBT, cognitive behavior therapy; ICBT, internet-based cognitive behavior therapy.

The participant registration process and study flow are shown in Figure 1. A total of 61 women met the inclusion criteria and were enrolled; 31 were allocated to the ICBT group, and 30 were allocated to the control group. Two participants assigned to the intervention group withdrew from the study before the intervention began; all 61 participants were included in the ITT analysis. The demographic data of the participants are presented in Table 1. Participants were predominantly young (mean [SD] age, 27.8 [9.0] years), had normal weight (mean [SD] BMI, 21.1 [3.6]), and had a mean (SD) duration of illness of 9.3 (8.8) years; half (31 [50.8%]) were employed. Statistical analyses revealed no significant differences in characteristics between groups (eTables 1 and 2 in Supplement 2).

**Table 1.** Participant Characteristics and Demographic Data at Baseline^a^

Characteristic	Intervention group (n = 31)	Control group (n = 30)
Age, mean (SD), y	28.3 (10.3)	27.2 (7.7)
BMI, mean (SD)	20.7 (3.9)	21.5 (3.3)
Years of education, mean (SD), y	13.7 (2.6)	14.0 (3.0)
Occupation status		
Employed	15 (48.4)	16 (53.3)
Student	7 (22.6)	5 (16.7)
Unemployed	9 (29.0)	9 (30.0)
Age at bulimia nervosa onset, mean (SD), y	19.2 (5.9)	17.9 (3.7)
Duration of illness, mean (SD), y	9.1 (9.2)	9.4 (8.5)
Marital status		
Single	23 (74.2)	24 (80.0)
Married	8 (25.8)	6 (20.0)
Divorced^b^	3 (9.7)	1 (3.3)
Alcohol consumption habit	13 (41.9)	12 (40.0)
Smoking habit	1 (3.2)	4 (13.3)
Family history of mental illness	8 (25.8)	9 (30.0)
Psychotropic medication use	14 (45.2)	14 (46.7)
Comorbidities^c^	14 (45.2)	14 (46.7)
Major depressive disorder	7 (22.6)	7 (23.3)
Dysthymic disorder	2 (6.5)	2 (6.7)
Bipolar disorder	1 (3.2)	1 (3.3)
Panic disorder	0	1 (3.3)
Agoraphobia	3 (9.7)	3 (10.0)
Social anxiety disorders	2 (6.5)	5 (16.7)
Obsessive-compulsive disorder	1 (3.2)	0
Generalized anxiety disorder	4 (12.9)	2 (6.7)

Abbreviation: BMI, body mass index (calculated as weight in kilograms divided by height in meters squared).

^a^ Unless indicated otherwise, values are presented as No. (%) of participants.

^b^ Indicates history of divorce.

^c^ Percentages sum to more than 100% because participants could have more than 1 comorbidity.

### Outcomes

#### Primary Outcome

Table 2 presents pretreatment and posttreatment outcomes for both groups. Based on the LMM analysis of the total sample, participants in the intervention group had a significant decrease (adjusted mean difference of 9.84 episodes [95% CI, 2.49-17.18 episodes]) in weekly combined frequency of binge eating and compensatory behavior episodes compared with the control group (*F*_1, 27.81_ = 7.54, *P* = .01; Cohen *d* = 0.73 [95% CI, 0.21-1.26]). Table 3 provides the adjusted mean difference and 95% CI for each clinical outcome.

**Table 2.** Pretreatment and Posttreatment Outcomes of Japanese Female Outpatients With Bulimia Nervosa by Guided Internet-Based Cognitive Behavior Therapy vs Usual Care^a^

Outcome	Intervention group	Control group
**Primary outcome**		
Combined weekly frequency of binge eating and compensatory behavior episodes^b^		
Pretreatment	19.13 (16.61)	14.27 (11.61)
Posttreatment	10.66 (12.35)	15.70 (14.17)
**Secondary outcomes**		
Weekly frequency of binge eating episodes		
Pretreatment	8.48 (7.38)	6.30 (5.96)
Posttreatment	5.41 (5.36)	6.57 (7.17)
Weekly frequency of compensatory behavior episodes		
Pretreatment	10.77 (9.77)	7.97 (6.54)
Posttreatment	5.24 (7.26)	9.13 (8.13)
EDE-Q global score		
Pretreatment	3.61 (1.23)	3.95 (0.97)
Posttreatment	2.76 (1.38)	3.75 (1.16)
PHQ-9 score		
Pretreatment	12.45 (6.04)	13.27 (6.37)
Posttreatment	12.07 (6.60)	12.33 (5.86)
GAD-7 score		
Pretreatment	7.10 (5.53)	8.47 (5.25)
Posttreatment	7.62 (4.67)	8.43 (4.96)
QALY calculated using the EQ-5D-5L		
Pretreatment	0.81 (0.14)	0.83 (0.10)
Posttreatment	0.81 (0.14)	0.83 (0.14)
BBQ score		
Pretreatment	28.58 (19.53)	28.63 (22.39)
Posttreatment	34.34 (22.93)	28.23 (20.73)

Abbreviations: BBQ, Brunnsviken Brief Quality of Life Scale; EDE-Q, Eating Disorder Examination Questionnaire; EQ-5D-5L, EuroQol 5 Dimensions 5 Levels; GAD-7, General Anxiety Disorder-7; PHQ-9, Patient Health Questionnaire-9; QALY, quality-adjusted life-year.

^a^ Values are presented as mean (SD). Posttreatment values represent 59 of the 61 participants because 2 participants in the intervention group did not complete the study.

^b^ Weekly frequency is reported as the No. of episodes.

**Table 3.** Bulimia Nervosa Severity as Estimated by the Mixed-Effects Model

Clinical value	Adjusted mean difference (95% CI)	*P* value	Effect size, Cohen *d* (95% CI)
Primary outcome			
Combined weekly frequency of binge eating and compensatory behavior episodes^a^	9.84 (2.49-17.18)	.01	0.73 (0.21-1.26)
Secondary outcomes^b^			
Weekly frequency of binge eating episodes	3.47 (0.26-6.67)	.04	0.56 (0.04-1.08)
Weekly frequency of compensatory behavior episodes	6.60 (1.40-11.80)	.01	0.83 (0.30-1.36)
EDE-Q global score	0.67 (0.20-1.14)	.006	0.67 (0.15-1.20)

Abbreviation: EDE-Q, Eating Disorder Examination Questionnaire.

^a^ Weekly frequency is reported as the No. of episodes.

^b^ Secondary outcome results were not adjusted for multiplicity and should be interpreted as exploratory.

#### Secondary Outcomes

The frequencies of both binge eating and compensatory behavior episodes significantly decreased in the ICBT group compared with the control group (Table 3). Participants allocated to the intervention group showed decreases in the frequency of binge eating episodes (adjusted mean difference, 3.47 episodes [95% CI, 0.26-6.67 episodes]; *P* = .04; Cohen *d* = 0.56 [95% CI, 0.04-1.08]) and compensatory behavior episodes (adjusted mean difference, 6.60 episodes [95% CI, 1.40-11.80 episodes]; *P* = .01; Cohen’s *d* = 0.83 [95% CI, 0.30-1.36]) compared with the control group. Furthermore, the EDE-Q global score significantly decreased by a mean of 0.67 points (95% CI, 0.20-1.14 points; *P* = .006; Cohen *d* = 0.67 [95% CI, 0.15-1.20]) in the intervention group compared with the control group. A detailed presentation of all secondary outcomes at each assessment time point is provided in eTable 3 in Supplement 2.

**Figure 2.**
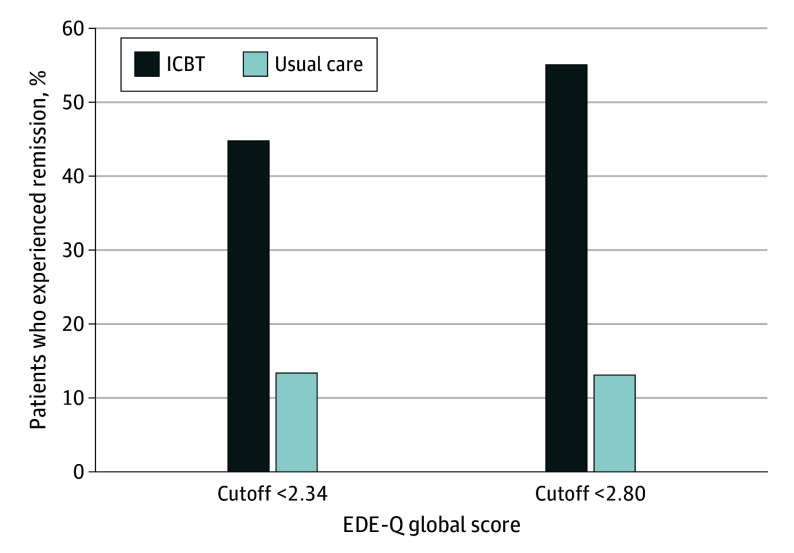
Participants in Remission in Each Group EDE-Q indicates Eating Disorder Examination Questionnaire; ICBT, internet-based cognitive behavior therapy.

Figure 2 illustrates the population of remitters in each group (detailed in eTable 4 in Supplement 2). At a cutoff value of 2.34 on the EDE-Q global score, remission rates were significantly higher in the intervention group (13 of 29 [44.8%]) compared with the control group (4 of 30 [13.3%]), with an OR of 5.28 (95% CI, 1.47-19.03; *P* = .01). Similarly, at a cutoff value of 2.80 on the EDE-Q global score, the intervention group demonstrated significantly higher remission rates (16 of 29 [55.2%]) compared with the control group (4 of 30 [13.3%]), with an OR of 8.00 (95% CI, 2.22-28.83; *P* < .001). However, no significant differences were observed between the groups in clinical symptoms other than eating disorders or in changes in BBQ score or QOL (eTable 3 in Supplement 2).

### Treatment Satisfaction and Module Completion

The mean (SD) WAI-SF therapeutic alliance score was 55.7 (18.2), indicating a moderate range. The mean (SD) CSQ score for treatment satisfaction was 22.8 (6.4), with 18 of 29 participants (62.1%) reporting high satisfaction and 21 of 29 (72.4%) stating that they would return to this treatment program if they needed help again (eTable 5 in Supplement 2). Among the 29 participants who received the intervention, most completed at least half of the treatment modules (15 [51.7%] completed at least 50%, and 7 [24.1%] completed >90%). Detailed completion rates for the guided ICBT program are provided in eTable 6 in Supplement 2.

### Sensitivity Analysis

The same results were obtained for the data with missing values imputed, reported in eTable 7 in Supplement 2, confirming the stability of the analysis. Similar results were also observed in the ANCOVA, further supporting the robustness of our findings (eTables 8 and 9 in Supplement 2). PPS analysis was conducted with 23 protocol-adherent participants in the intervention group and yielded results consistent with the ITT analysis, with a comparable effect size for the primary outcome (Cohen *d* = 0.81 [95% CI, 0.24-1.37]) (eResults and eTable 10 in Supplement 2). Observed power values were calculated for reference and are provided in the eResults in Supplement 2.

### Assessment of Blinding

There was no evidence suggesting that blinding was compromised. These results are provided in the eResults and in eTable 11 in Supplement 2.

## Discussion

In this multicenter RCT conducted in Japan for women with BN, guided ICBT significantly alleviated eating disorder symptoms. The minimal amount of missing data, together with the results of the sensitivity analyses, supports the primary findings.

Our findings demonstrate that guided ICBT shows promise in alleviating BN. To our knowledge, this RCT is the first study in East Asia to demonstrate these effects, expanding the evidence base for ICBT in the treatment of eating disorders.^51,52^ The effect sizes (Cohen *d* = 0.56-0.83) and remission rates (44.8%-55.2%) in the current study were comparable to those reported previously for face-to-face CBT (Hedges *g* = 0.52-0.88; remission rates: 34.6%-42.1%).^53,54^ Our findings additionally indicate that guided ICBT is effective in reducing compensatory behaviors in women with BN, a discovery that contradicts a previous German RCT.^14^ This discrepancy may be attributed to the unique components based on our CBT model, which includes cue exposure to strengthen resistance to urges to engage in compensatory behaviors and body image rescripting. Given the complexity of CBT, further research is warranted to investigate the effects of specific CBT components.

In the current RCT, therapists responded within 24 hours, and more than half of our intervention participants (18 of 29 [62.1%]) expressed satisfaction with the treatment. Satisfaction with guided ICBT was reported by 78% (54 of 91) of the participants in a previous RCT, with participants particularly appreciating the added value of therapist support.^16^ These findings suggest that ICBT, including therapist support and responding to patients as soon as possible, is well accepted.

The aforementioned German RCT^14^ found that guided ICBT did not lead to significant changes in comorbid symptoms (eg, depression and anxiety) between groups, which is consistent with our results. That is, the specific CBT techniques that focused on BN did not have a significant indirect effect on depressive symptoms. A network meta-analysis suggested that behavioral activation is a beneficial component of CBT for addressing depression.^55^ Neither the current RCT conducted in Japan nor the previous RCT performed in Germany included the behavioral activation component. Given the high lifetime prevalence of major depressive disorder among individuals with BN (approximately 61%) and the potential impact of anxiety and depressive symptoms on eating disorder behaviors,^9,56^ it may be worthwhile to implement optional modules targeting anxiety and depression in ICBT programs for individuals with BN.

### Limitations

This study has a few limitations. Most of the participants were recruited through online advertisements, and they are likely part of the population with higher information and communication technology (ICT) literacy. This limitation may hinder the generalizability of the findings; however, as of October 2024, 5.52 billion people—67.5% of the global population—are internet users.^57^ In regions with well-developed ICT infrastructure, such as northern Europe, North America, and East Asia, including Japan and Korea, nearly all individuals have access to the internet. Thus, our findings would likely be reflected in most women with BN. Further, follow-up assessments were not conducted in this study, and the long-term effectiveness of the intervention remains unclear.

## Conclusions

The findings of this RCT suggest that guided ICBT may alleviate the severity of BN and increase remission when implemented in a UC setting, and guided ICBT would likely be well accepted by women with BN. ICBT approaches could improve access to effective treatments for BN, which are currently limited.

## Notes

Supplement 1.
Trial Protocol

Supplement 2.
eMethods
**eFigure.** User Interface of the Bulimia Nervosa Internet-Based Cognitive Behavior Therapy Program **eTable 1.** Comparison of Baseline Characteristics Between Groups **eTable 2.** Baseline Outcome Comparison in Bulimia Nervosa Treatment: Intervention vs Control Groups **eTable 3.** Secondary Outcomes by Study Group Over Time **eTable 4.** Remission Rates and Odds Ratios for Each Group Based on the 2 Criteria **eTable 5.** Posttreatment Outcomes: Therapeutic Alliance and Client Satisfaction **eTable 6.** Treatment Adherence in the Intervention Group **eTable 7.** Clinical Variables by Study Group Over Time With Imputed Missing Values **eTable 8.** Comparison of Clinical Variables Using Analysis of Covariance **eTable 9.** Group Comparisons by Analysis of Covariance After Missing Data Imputation **eTable 10.** Per-Protocol Set (PPS) Analysis: Outcomes by Study Group Over Time
eResults
**eTable 11.** Results of Blinding Assessment
eReferences

Supplement 3.
Data Sharing Statement
