# Supplementary material for: Guided Internet-Based Cognitive Behavior Therapy for Women With Bulimia Nervosa: A Randomized Clinical Trial
Source: JAMA Netw Open. 2025 Aug 5;8(8):e2525165. doi: 10.1001/jamanetworkopen.2025.25165 (PMC12326282; doi:10.1001/jamanetworkopen.2025.25165)
Supplement: Supplement 1. — Trial Protocol [file jamanetwopen-e2525165-s001.pdf]

Effectiveness of Internet-Based Cognitive Behavioral Therapy for Bulimia Nervosa: A Randomized Control Trial

Trial Protocol

Sayo Hamatani[1,2,3]; Kazuki Matsumoto[4]; Gerhard Andersson[5,6,7] et al.

[1] Research Center for Child Mental Development, University of Fukui, Fukui, Japan

[2] Division of Developmental Higher Brain Functions, United Graduate School of Child Development, University of Fukui, Fukui, Japan

[3] Department of Child and Adolescent Psychological Medicine, University of Fukui Hospital, Fukui, Japan

[4] Division of Clinical Psychology, Kagoshima University Hospital, Kagoshima, Japan

[5] Department of Behavioural Sciences and Learning, Linköping University, Linköping, Sweden

[6] Department of Biomedical and Clinical Science, Linköping University, Linköping, Sweden

[7] Department of Clinical Neuroscience, Karolinska Institute, Stockholm, Sweden

Sections of this document have been published here: Hamatani S, Matsumoto K, Andersson G, Tomioka Y, Numata S, Kamashita R,Sekiguchi A, Sato Y, Fukudo S, Sasaki N, Nakamura M, Otani R, Sakuta R, Hirano Y,Kosaka H, Mizuno Y. Guided Internet-Based Cognitive Behavioral Therapy for Women With Bulimia Nervosa: Protocol for a Multicenter Randomized Controlled Trial. *JMIR Res Protoc*. 2023;12:e49828.
doi: 10.2196/49828

1. Study Overview Based on the PICO Framework

Criteria	Assessments
**P:** Population	*Inclusion criteria:*<br>(1) Women aged 13-65 years and diagnosed with BN according to DSM-5 criteria during a clinical interview.<br>(2) Having a BMI over 17.5 kg/m2<br>(3) Using computers, tablets, smartphones, etc, on a daily basis, with access to the internet and the minimum necessary information and communications technology skills<br>(4) No history of CBT in the last 2 years<br>*Exclusion criteria:*<br>(1) Serious mental disorders such as organic brain disorders, psychotic disorders, and drug dependence<br>(2) Imminent risk of suicide<br>(3) Repeated engagement in antisocial behavior<br>(4) Serious progressive physical disease<br>(5) Difficulty in exposure to feared objects due to severe stress reactions or dissociation symptoms due to acute stress disorder or posttraumatic stress disorder
**I:** Intervention	● 12 weeks of a web-based intervention with therapist's guide for bulimia nervosa
**C:**<br>Control	● 12 weeks waiting time
**O:**<br>Outcome	*Primary Confirmatory*<br>● Number of binge eating and compensatory behavior episodes<br>*Secondary Confirmatory*<br>● Number of binge eating episodes<br>● Number of compensatory behavior episodes<br>● Global eating psychopathology (EDE-Q)<br>● Comorbid depression (PHQ-9)<br>● Comorbid anxiety (GAD-7)<br>● Quality of life (EQ-5D-5L)<br>● Well-being (BBQ)<br>*Additional Assessments*<br>● Working alliance in the treatment (WAI-SF)<br>● Satisfaction for the treatment (CSQ)

2. Introduction

Bulimia nervosa (BN) is an eating disorder characterized by recurrent episodes of binge eating followed by compensatory behaviors, such as purging or excessive exercise, and a persistent preoccupation with body shape and weight.[1] Individuals with BN often experience body image distortion, perceiving their bodies as larger or heavier than they actually are, despite maintaining a normal or even underweight body weight.[2] This distorted body image drives the compulsive engagement in purging, fasting, or excessive exercise to alleviate perceived physical flaws.[3] Body image distortion encompasses both perceptual disturbances, such as misperceptions of body size and shape, and cognitive distortions, such as negative body image and dissatisfaction.[4] Repeated cycles of binge eating and compensatory behaviors can lead to significant physical and psychological health consequences. Purging behaviors, particularly self-induced vomiting, can result in a range of physical complications, including dental erosion, electrolyte imbalances, esophageal tears, and salivary gland enlargement.[5,6] From a psychological perspective, individuals with BN often experience a host of mental health issues, such as low self-esteem, self-harm, suicidal ideation, depression, anxiety disorders, sleep disturbances, and chronic fatigue.[7-12]

Epidemiological studies have consistently demonstrated a high prevalence of eating disorders, particularly among women. In Western countries, approximately 2.6% of women are affected.[13] Moreover, the overall prevalence of eating disorders has shown a significant increase, rising from 3.5% between 2000 and 2006 to 7.8% between 2013 and 2018.[14] A recent meta-analysis estimated a lifetime prevalence of eating disorders of 0.91% and a 12-month prevalence of 0.4%, with a lifetime prevalence of BN specifically at

0.6%.[13] While face-to-face cognitive-behavioral therapy (CBT) has been established as an effective treatment for eating disorders,[15] limited access to such treatment remains a significant challenge.[16] The limited accessibility of treatment for eating disorders is a significant concern. Despite the prevalence of these conditions, only 19-36% of individuals with eating disorders initiate treatment within the first year,[17-19] and a substantial proportion (35-40%) do not receive evidence-based care.[20,21] Furthermore, the average delay in seeking treatment is a protracted 5-15 years.[16,22] Barriers to accessing adequate treatment, such as geographic limitations, provider shortages, and stigma, have hindered the care of individuals with eating disorders. Web-mediated interventions have emerged as a potentially effective solution, particularly for early intervention, by overcoming many of these challenges.[23,24]

Among CBT interventions for BN, enhanced CBT (CBT-E) proposed by Fairburn has been the most extensively studied.[25] CBT-E has demonstrated efficacy in the treatment of eating disorders, including BN. By promoting the early adoption of healthy eating behaviors and facilitating both behavioral and cognitive modifications through psychoeducation and self-monitoring, enhanced CBT offers a comprehensive approach to recovery.[26] Even after the establishment of CBT-E, promising cognitive-behavioral techniques for BN continue to be developed. In the currently, several other CBT-based interventions may also prove effective in treating BN. These include attentional bias modification training to redirect excessive focus on body shape, weight, and food,[27] relaxation and mindfulness techniques to alleviate physiological arousal, such as anxiety and tension,[28] trauma-focused therapy to address experiences of body-related criticism,[29] and impulse control training, such as cue exposure.[30] Furthermore, network analysis suggests that fear of weight gain is a

core feature of BN psychopathology, while hypersensitivity to physical sensations may link BN to anxiety and depression.[31] Based on this evidence, the meta-analysis posits that exposure therapy targeting fear of weight gain and interoceptive exposure interventions are effective treatments for BN.

Our research team has published a comprehensive therapist's manual outlining the key components of CBT for BN.[32] Our research group has recently developed a web-based intervention based on this manual, carefully considering its cultural applicability within a Japanese context.[33]

3. Study Design

This study is designed as a multicenter, prospective, randomized, assessor-blinded clinical trial. The RCT and follow-up study are planned from August 2022 to October 2026 (UMIN000048732). The current RCT is conducted at the following 7 institutions (6 university hospitals and a national medical center) in Japan: University of Fukui Hospital, Kagoshima University Hospital, Chiba University Hospital, Tokushima University Hospital, Dokkyo Medical University, Tohoku University Hospital, and National Center for Neurology and Psychiatry. This research protocol and procedures of the current RCT have been reviewed and approved by the Ethics Committee at the University of Fukui (Approval Number: 20220054). Participants who qualify for the study, based on screening and a diagnostic interview, are randomly assigned to one of two groups: an Intervention group, with immediate access to a guided ICBT program for BN; or a control group (Waitlist), with delayed access to the intervention.

4. Objective and Hypotheses

4.1.1. Primary Hypotheses

This study aims to evaluate the effectiveness of therapist-guided ICBT for women with BN in a Japanese clinical setting. We hypothesize that the addition of guided ICBT to usual care (UC) will yield superior outcomes compared to UC alone.

4.1.2. Secondary Hypotheses

We also hypothesize that culturally adapted guided ICBT will be well-accepted.

5. Method

5.1. Intervention Group

Participants allocated to the intervention group are recommended to engage in a 12-week therapist-guided ICBT program. This ICBT program, consisting of 12 sessions, is based on a treatment model focused on psychoeducation about BN and modification of characteristic cognitive-behavioral patterns.[32] Participants in this group receive weekly reminder emails to complete the ICBT program. The ICBT program includes videos, text, illustrations, and diagrams to facilitate self-guided CBT practice. The participants are able to access the ICBT program through their own devices. It is expected that each session can be completed within 15 minutes. Table 1 provides a description of the treatment modules and CBT techniques implemented in the ICBT program. At the end of each session, participants are assigned homework, requiring them to practice the learned CBT

skills tailored to their specific BN characteristics and report back to their therapist. Participants in the intervention group can consult with their therapist at any time via the chat system regarding any aspect of BN treatment or the ICBT program. The therapist (SH, the first author) is an experienced expert in CBT for BN and one of the developers of the face-to-face CBT manual that serves as the foundation for this ICBT program. A secure web-based platform is used for communication between therapists and participants, distribution of program materials, and collection of assessments.[34]

Table 1. Description of Treatment Modules and CBT Techniques for Each Session

Session No.	Module and Description
0	Assessment<br>An explanation of the program's usage and structure will be provided, followed by the collection of basic clinical background data including family composition and chief complaints. Treatment goals will then be established.
1	Psychoeducation about BN and the CBT model<br>The study provides an overview of the DSM-5 TEXT REVISION diagnostic classification and the epidemiological features of BN.[1,35] Psychoeducational aspects of CBT for BN are introduced. A graphical representation illustrates the vicious cycle encompassing perception, attention, imagery, emotion, memory, thought, and habitual behavior.[32]
2	Relaxation and mindfulness meditation<br>The module will offer a range of relaxation techniques, such as mindfulness meditation, which will include education on the autonomic nervous system, deep breathing exercises, progressive muscle relaxation, and mindfulness meditation training.[28,30]

3	Meta-cognition training<br>Metacognition, a cognitive function, has been found to be impaired in individuals with eating disorders,[36] and research has shown that cognitive impairments can negatively impact QOL.[37] Meta-cognition training hold promise for improving the severity of eating disorders, depressive symptoms, and QOL.[24,38] These interventions typically target cognitive distortions such as dichotomous thinking, emotional reasoning, jumping to conclusions, and perfectionism.[24,32]
4	Modification of attention bias and body-image<br>Individuals with BN exhibit a heightened attentional bias towards body shape, weight, and overeating.[39,40] Training to redirect attention from food-related stimuli to neutral stimuli is provided.[41,42] Individuals with BN frequently engage in negative self-evaluation of their body shape and weight.[43] Through recognition of the threatening bias towards their natural body shape and weight, the individuals could employ relaxation and metacognitive techniques to address perceived concerns regarding their body shape and weight.
5	Behavior experiment<br>Behavioral experiments are used to confirm that eating does not lead to uncontrollable binge eating or catastrophic weight gain.[44] The first step involves observing whether eating a small amount of any food triggers a binge episode. Next, they experiment to see if these eating episodes lead to as much weight gain as they fear.
6	Fostering the establishment of healthy eating habits<br>Self-monitoring of dietary intake through food diaries has been demonstrated to significantly reduce binge eating episodes within the context of web-based interventions.[45] Our proposed intervention utilizes Japanese cuisine to facilitate the attainment of a balanced dietary pattern.[32] This approach was developed based on

	nutritional rehabilitation, termed "mechanical diet" by Garner et al. (2017).[46]
7	Anxiety hierarchies and gradual exposure<br><br>Addressing fears related to overeating may reduce binge eating in BN.[47,48] Participants were asked to identify specific foods, cooking methods, and menu items that they feared and to rate their fear on a scale of 0-100. A hierarchy of fears was then created, and participants were encouraged to expose themselves to the least fear-provoking items first. The importance of avoiding compensatory behaviors was also emphasized.
8	Cue exposure for binge eating and compensatory behavior<br><br>Triggers for binge eating and compensatory behavior vary from person to person, but exposure to these triggers may reduce binge eating and compensatory behaviors.[30] For example, participants were encouraged to sit in front of binge eating triggers, such as specific foods or the refrigerator, and tolerate the urge to binge for a period of time.
9	Reconstructing negative self-talk<br><br>Negative self-talk related to eating is frequently observed in BN.[49] Such self-talk can shape one's identity and self-worth.[50] Cognitive restructuring interventions aim to promote the development of alternative, more adaptive cognitive appraisals of these self-statements.
10	Imagery Rescripting<br><br>Many individuals with BN have experienced negative childhood experiences.[51] This module is designed for individuals who have developed negative self-schemas as a result of their interpretation of traumatic experiences. This module helps the individual reframe the painful memories associated with your trauma into something less upsetting.

11	Rewriting residual irrational beliefs, schema work<br>By rewriting irrational/dysfunctional beliefs (schemas), it may be possible to reduce the frequency of binge eating and compensatory behavior, as well as concerns about body shape.[52] Irrational/Dysfunctional beliefs are identified and replaced with alternative beliefs.
12	Relapse prevention.<br>Participants are asked to summarize what they have learned from the treatment and to use this summary to plan for future actions to further reduce symptoms and prevent relapse.

5.2. Control Group

Participants assigned to the control group are encouraged to continue their usual care (UC) at a Japanese psychiatric clinic on a regular basis. UC group participants are also informed that they will have the opportunity to receive the guided ICBT program in the future by being placed on a waiting list. Control group participants are able to continue with counseling, antipsychotics, or other pharmacotherapy as needed, in consultation with their primary care physician, based on their individual condition and needs. However, as a general rule, it is prohibited to start or change any secondary therapies such as medication or counseling during the study period. Participants are asked to immediately report to the study office if there are any additions or discontinuations of medication therapy in unavoidable circumstances. It is planned that all changes will be documented in writing, along with the reasons.

5.3. Participant Recruitment

Participants are being recruited from August 2022 to July 2024, through flyers posted at our joint research facility and online advertisements (e.g., Google Ads, Twitter, and Facebook), as well as newspaper advertisements. Participants must be currently receiving treatment at a psychiatric hospital and must provide a diagnosis certificate for BN from their attending physician. It is stated that participants will be responsible for their own usual care (UC) costs during the study period. Additionally, all participants, regardless of their assigned group, will receive a 12,000 JPY (76.02 USD, 1 USD = 157.855 JPY as of 30-Dec-2024) stipend upon completion of the study.

5.4. Inclusion and Exclusion Criteria

Inclusion Criteria

(1) Women aged 13-65 years and Diagnosed with BN according to DSM-5 criteria during a clinical interview.

(2) Having a BMI over 17.5 kg/m2.

(3) Using computers, tablets, smartphones, etc, daily, with access to the internet and the minimum necessary information and communications technology skills.

(4) No history of CBT in the last 2 years.

Exclusion criteria:

(1) Serious mental disorders such as organic brain disorders, psychotic disorders, and drug dependence.

(2) Imminent risk of suicide.

(3) Repeated engagement in antisocial behavior.

(4) Serious progressive physical disease.

(5) Difficulty in exposure to feared objects due to severe stress reactions or dissociation symptoms due to acute stress disorder or posttraumatic stress disorder.

5.5. Randomization and Blinding

An independent data management team at Fukui University randomizes all participants to either the intervention or control group using the UMIN Medical Research Support (Case Registration and Allocation) System Cloud (UMIN System Cloud) and employs forced balanced randomization. This randomization sequence is concealed from both participants and their primary care physicians to ensure impartiality. Furthermore, an independent evaluation team at Fukui University remains blinded to the group assignment of the participants being evaluated to maintain the integrity of the study outcomes.

5.6. Measures

Potential participants who apply for the study provide their name, age, sex assigned at birth, height, recent weight, educational background, employment history, employment status, marital status, family history of mental illness, medical history, current medications and dosages, and information about the devices (smartphone, personal computer [PC], tablet PC, etc.) they are using for the study through an online data

collection system.

5.6.1. Eligibility Screening and Structured Clinical Interviews

Eligibility assessments are conducted by experienced clinicians or researchers via face-to-face, telephone, or video conference interviews. A semi-structured diagnostic interview, the Mini-International Neuropsychiatric Interview (M.I.N.I.), is administered to assess for the presence of comorbid conditions.[53,54]

5.6.2. Outcomes

The study assesses various outcomes, such as eating disorder severity, comorbid depression and anxiety, quality of life (QOL), satisfaction with the treatment, and the therapeutic alliance.

5.6.3. Primary Outcome

The primary outcome, the weekly combined frequency of binge eating and compensatory behavior episodes, is assessed at baseline and 12 weeks post-intervention. The independent assessor team at the University of Fukui measures the primary outcome via telephone.

5.6.4. Secondary Outcomes

- Weekly frequency of binge eating episodes: The independent assessor team askes the weekly frequency of binge episodes via telephone.
- Weekly frequency of compensatory behavior episodes: The independent assessor team askes the weekly frequency of compensatory behavior episodes via telephone.

5.6.5. Eating Disorder Examination Questionnaire 6.0, EDE-Q: The EDE-Q is a widely used self-report instrument for assessing the severity and frequency of symptoms associated with eating disorders.[55] It comprises 28 items that evaluate eating behaviors, attitudes, and concerns over the last 4-week period. Responses are rated on a 7-point Likert scale, ranging from 0 (never) to 6 (daily). The EDE-Q is composed of four subscales: restraint, eating concern, shape concern, and weight concern. The Japanese version of the EDE-Q has demonstrated adequate internal consistency and good reliability and validity for these four factors.[56] The severity of each subscale is represented by the total score for that subscale, and the EDE-Q global score is derived from the average of the subscale scores. Among a sample of 727 Japanese female university students (age: M = 19.32, SD = 1.08; BMI: M = 20.46, SD = 2.56), the mean (SD) EDE-Q global score was 1.51 (1.02). Mean (SD) scores for the restraint, eating concern, shape concern, and weight concern subscales were 0.86 (1.10), 0.58 (0.82), 2.45 (1.52), and 2.13 (1.41), respectively (Mitsui, 2013). In the present study, the EDE-Q global score is designated as secondary outcomes.

5.6.6. **Secondary Exploratory Outcomes**

- **The Patient Health Questionnaire 9 items (PHQ-9):** The PHQ-9 is a brief self-report measure designed to screen for major depressive disorder (MDD). It is based on the diagnostic criteria for MDD as outlined in the DSM-IV and assesses the severity of depressive symptoms.[57,58] A diagnosis of MDD is supported if five or more of the nine DSM-IV criteria for MDD have been present for at least half of the days during the past two weeks and at least one of the symptoms is either depressed mood or

loss of interest or pleasure. The PHQ-9 consists of nine items rated on a 4-point Likert scale, with a total score range of 0 to 27. The interpretation of PHQ-9 scores is categorized into five levels of severity: minimal (0-4), mild (5-9), moderate (10-14), moderately severe (15-19), and severe (20-27). The psychometric properties of the Japanese version of the PHQ-9 have been well-established.[59]

- **The Generalized anxiety disorder 7 item (GAD-7):** The GAD-7 is a brief self-report measure designed to screen for generalized anxiety disorder (GAD). It is based on the diagnostic criteria for GAD outlined in the DSM-IV and assesses the severity of GAD symptoms.[60] The GAD-7 consists of seven items rated on a 4-point Likert scale, with a total score range of 0 to 21. A cutoff score of 10 or higher is commonly used to identify individuals with probable GAD. The interpretation of GAD-7 scores is categorized into four levels of severity: minimal (0-4), mild (5-9), moderate (10-14), and severe (15-21). The psychometric properties of the Japanese version of the GAD-7 have been well-established.[59]
- **The EuroQol 5 dimensions 5-level (EQ-5D-5L):** The EQ-5D-5L is a quality-of-life measure that assesses five dimensions of health: mobility, self-care, usual activities, pain/discomfort, and anxiety/depression.[61] Responses are rated on a 5-point Likert scale ranging from 1 (no problems) to 5 (extreme problems). A health index score can be derived from the EQ-5D-5L, ranging from 0 (death) to 1 (full health). A Japanese valuation of EQ-5D-5L was developed to create the QOL value for a quality of adjusted life year using a national sample of 1,026 individuals.[62]
- **The Brunnsviken Brief Quality of Life Scale (BBQ):** The BBQ was developed to measure subjective QOL. It consists of 12 items covering six life areas: Leisure, View on life, Creativity, Learning, Friends

and Friendship, and View on self.[63] For each life area, respondents rate their satisfaction and importance on a 5-point Likert scale ranging from 0 (strongly disagree) to 4 (strongly agree). The total BBQ score can range from 0 to 96. The validity of the Japanese version of the BBQ has been demonstrated by our research group.[64]

5.6.7. Other Measures

- **The 8 item version of Client Satisfaction Questionnaire (CSQ-8):** The CSQ is a commonly used questionnaire for assessing satisfaction with psychotherapy services. While the original CSQ consists of 18 items, the 8-item version of CSQ (CSQ-8) has also demonstrated high internal consistency, and satisfaction scores measured by these scales have been shown to predict positive outcomes.[65] The reliability and validity of the Japanese version of the CSQ-8 have been established.[66]
- **The Working Alliance Inventory-Short Form (WAI-SF):** The WAI-SF is a 12-item measure that assesses the strength of the bond between the patient and therapist, as well as the degree of agreement on treatment goals and content.[67] Each item is rated on a 7-point Likert scale. The total WAI-SF score ranges from 12 to 84, with higher scores indicating a stronger therapeutic alliance. The Japanese version of the WAI-SF has demonstrated good reliability and validity.[68]

6. Planned Statistical Analysis

Statistical analyses will be performed using SPSS Statistics software (version 29; IBM Corp) or R Statistics.[69] Analyses will be conducted based on the intention-to-treat principle (ITT), in accordance with the CONSORT

2010 checklist.[70] All *P*-values will be two-tailed, and a P-value < .05 will be considered statistically significant.

6.1. Primary Confirmatory Analyses

The primary indicator for evaluating the effectiveness of the intervention will be whether the difference between the intervention and control groups at 12 weeks is statistically and clinically significant. The primary outcome will be analyzed as the total number of binge eating and purging episodes. A mixed linear model (MLM) will be constructed to examine the effects of time, group (intervention or control), and their interaction. The treatment effect will be calculated as a standardized effect size using Cohen's *d* and will also be presented as the least squares mean difference estimate with a 95% confidence interval (95% CI).

The protocol originally specified analysis of covariance (ANCOVA) as the primary analysis method, as outlined in the published protocol paper. However, to better align with the evolving understanding of data characteristics and to enhance analytical precision, we updated the protocol and adopted linear mixed models (LMM) as the primary analysis method, following recommendations from prior studies.[71,72] Analysis of covariance (ANCOVA) will be reported as a sensitivity analysis.

6.2. Secondary Confirmatory Analyses

Secondary analyses will be conducted using the same statistical model as the primary analysis to evaluate the multifaceted effects of the intervention through secondary outcomes (eating disorder severity, depression,

anxiety, and quality of life). The analyses will examine differences between groups as well as changes over time.

6.3. Exploratory Analyses and Additional Statistical Tests

To assess treatment satisfaction and therapeutic alliance, the post-treatment mean values will be calculated for the intervention group. To evaluate baseline differences between the two groups, independent samples t-tests, chi-square tests, and Fisher's exact tests will be used. For the binary outcome of remission in bulimia nervosa (BN), a chi-square test will be employed to assess the significance of the odds ratio (OR). The criteria for remission will be determined using the cutoff values of the EDE-Q score, 2.34 or 2.80.[73,74]

6.4. Missing Data and Sensitivity Analyses

Missing data will be imputed using Multiple Imputation by Chained Equations (MICE). For sensitivity analyses, the difference in mean change scores from baseline to 3 months between the two groups will be examined using analysis of covariance (ANCOVA) to determine statistical significance. Additionally, observed power will be calculated to assess the validity of the primary results. A per-protocol set (PPS) analysis will also be conducted by excluding patients who deviated from the study protocol.

6.5. Assessment of Blinding

To evaluate whether the blinding of the assessment was successful, the Bang's method and James's method

were applied.[75,76] After the post-assessment, independent evaluators were asked, "Which treatment do you think the participant received?" The response options provided were "Guided ICBT," "UC," or "Don't know."

Bang's blinding index ranges from -1 to 1, where 0 indicates perfect blinding. A value of 1 indicates complete unblinding, while -1 suggests that all participants' treatment allocations were incorrectly guessed. If the one-sided confidence interval excludes 0, the study is considered to have insufficient blinding.

Additionally, the James blinding index was calculated to provide further insight into the success of blinding. The James index is a continuous measure ranging from 0 to 1, where a score of 0.5 indicates perfect blinding. Higher values suggest a greater deviation from perfect blinding. Confidence intervals for the James index were also calculated to assess the adequacy of blinding.

6.6. Sample Size Calculation

The target sample size for this study is set at 60 participants, with 30 participants in each group. The rationale for this sample size was determined using the statistical analysis software G*Power 3.1,[77] based on a calculation for an independent t-test. The effect size (Cohen's d = 0.9) was derived from a previous study on ICBT for bulimia nervosa, which reported significant reductions in the frequency of binge eating and purging behaviors compared to the waitlist group.[78] Assuming an expected effect size of 0.9 for this study, with a significance level of 0.05 (two-tailed) and a power of 80% (1-β), a minimum of 21 participants per group is required.

To account for a dropout rate of 30%, an additional 9 participants per group are anticipated, bringing the required sample size to 30 participants per group. Therefore, the total target sample size for the study is 60 participants. This ensures sufficient power to detect significant differences between the intervention and control groups.

7. Discussion and Conclusion

In this study, we will conduct the first randomized controlled trial (RCT) to evaluate the effectiveness of therapist-guided internet-based cognitive behavioral therapy (ICBT) for Japanese female patients with bulimia nervosa (BN). While a recent study conducted in the Netherlands included participants with a wide BMI range (19.5–40.0 kg/m^2) and mixed diagnoses,[79] this study adopts focused eligibility criteria, specifically targeting patients with bulimia as the diagnosis and excluding other eating disorders. This approach ensures a more homogeneous study population, thereby enhancing the reliability of treatment effect estimates. A systematic review and meta-analysis of e-therapy for eating disorders highlighted promising improvements in symptoms such as binge eating, vomiting, and laxative misuse but found inconclusive evidence regarding its impact on overeating frequency in BN patients due to heterogeneity in study quality.[80] Recent single-arm studies of ICBT have reported positive outcomes,[81] yet RCTs remain the gold standard for clinical trials, providing the highest level of evidence.[82] Previous studies that included BN patients have not provided effect estimates exclusively for BN, often grouping them with other eating disorders.[83,84] This RCT addresses this gap by offering insights into the effectiveness of guided ICBT in reducing binge eating and purging frequency and addressing secondary

symptoms in BN patients.

Additionally, this study is the first to explore the effects of ICBT on eating behavior changes in Japan, accounting for the country’s unique food culture and healthcare system. By adopting a multicenter design, the study aims to minimize bias and improve the generalizability of its findings, providing culturally relevant evidence that could guide future interventions.

This RCT seeks to validate a Japanese culture–adapted ICBT program for BN and contribute to the limited evidence base regarding its efficacy. Given the low implementation rate of CBT in Japanese psychiatric clinics (6.2%) (Takahashi et al.,2018),[85] this research could pave the way for broader access to early treatment for BN patients. Positive results from this trial could lead to improvements in managing eating disorders, which often become chronic and challenging to treat.

8. Declarations

8.1. Publication Rules

All researchers involved in this study comply with the "Declaration of Helsinki (2013 Fortaleza Revision)" and the "Ethical Guidelines for Life Sciences and Medical Research Involving Human Subjects (Notice No. 1 of the Ministry of Education, Culture, Sports, Science and Technology, the Ministry of Health, Labour and Welfare, and the Ministry of Economy, Trade and Industry, dated March 23, 2021)." All members of the research team who contributed to the development and implementation of this study by supporting data collection, analysis, interpretation, or publication of the study will be credited as authors. Each author is given sufficient time to

review the manuscript and the opportunity to approve it. This study is pre-registered at https://www.umin.ac.jp/ (UMIN000048732).

8.2. Ethics Approval an Informed Consent

This trial protocol is approved by the Ethics Committee for Medical Research at the University of Fukui (Approval Number: 20220054). Written informed consent is obtained from all participants, and the study is conducted in accordance with the Declaration of Helsinki and good clinical practice guidelines.

8.3. Individual Benefits, Burdens, and Risks Associated with Study Participation

Participants will have free access to the Internet Cognitive Behavioral Therapy (ICBT) program for 12 weeks. Participants will be responsible for the cost of TAU, but the guided ICBT will be free of charge. Participants will receive a compensation of 12,000 yen after completing the intervention or waitlist, regardless of group assignment. This includes the length of time the patient spent on the study and the difficulty of patient recruitment. To participate in this study, a referral letter from some hospitals is required, and participants may be responsible for the cost. Data collection and payment of compensation will be conducted by the research staff of Fukui University.

The content of the diagnostic interview and modules may cause negative emotions or feelings of fatigue in participants. For this reason, participants are informed that they have the right to pause or end the study at

any time, and that they will not be disadvantaged in any way. In addition, exclusion criteria are set to ensure that individuals in an acute stress state do not participate in ICBT, and strict checks are carried out before the study begins. Furthermore, in preparation for psychological stress, all participants are provided with the contact details of the research staff at Fukui University, and a system is in place to ensure that they can receive support if necessary. During the research period, all participants (waiting list group and intervention group) can continue to receive outpatient psychotherapy, medical treatment, and other treatment that they have been receiving up to this point. This research will be conducted online, but since the participants are those who are already regularly visiting medical institutions, including those who live far away, there is a system in place that allows them to promptly visit a nearby doctor if necessary.

8.4. Statement on Medical Justifiability

This study has been approved by the Ethics Review Committee of Fukui University (Approval Number: 20220054) and is conducted in accordance with the Declaration of Helsinki and the "Ethical Guidelines for Life Sciences and Medical Research Involving Human Subjects." Furthermore, the study design and implementation methods have been deemed medically and psychotherapeutically appropriate based on the opinions of certified clinical psychologists and medical professionals.

8.5. Competing Interests and Funding

This study is supported by a JSPS Grant-in-Aid for Scientific Research (23K22256) and a research grant from the Lotte Foundation. Additionally, it has been reported to the Clinical Research Conflict of Interest Review Committee of Fukui University that no special benefits, such as research funding, salaries, or honoraria, have been received from any specific companies or organizations in connection with this study, and it has been determined that there are no conflicts of interest. The study will be conducted fairly, ensuring that participants are not subjected to any disadvantages and that the research results are not distorted in any way.

9. Timeline

- **Protocol finalization date**: June 22, 2022
- **Ethics committee approval dat**e: August 15, 2022
- **Trial public registration date（UMIN）**: August 23, 2022
- **Enrollment start date**: August 24, 2022
- **Protocol publication date**: September 19, 2023 (published in *JMIR Research Protocols*)
- **Recruitment completion date**: July 31, 2024
- **Intervention completion date**: October 31, 2024
- **Data collection and registration completion date**: November 30, 2024
- **Analysis completion date**: December 30, 2024

References

1. American Psychiatric Association. *Diagnostic and statistical manual of mental disorders* (5th ed., text rev.). Washington, DC: American Psychiatric Association Publishing; 2022. https://www.mredscircleoftrust.com/storage/app/media/DSM 5 TR.pdf

2. Flett GL, Newby J, Hewitt PL, Persaud C. Perfectionistic automatic thoughts, trait perfectionism, and bulimic automatic thoughts in young women. *J Rat-Emo Cognitive-Behav Ther.* 2011;29(3):192-206. https://doi.org/10.1007/s10942-011-0135-3

3. Kaye W. Neurobiology of anorexia and bulimia nervosa. *Physiol Behav.* 2008;94(1):121-135. https://doi.org/10.1016/j.physbeh.2007.11.037

4. Yamamotova A, Bulant J, Bocek V, Papezova H. Dissatisfaction with own body makes patients with eating disorders more sensitive to pain. *J Pain Res*. 2017;*10*:1667-1675. https://doi.org/10.2147/JPR.S133425

5. Spalter AR, Gwirtsman HE, Demitrack MA, Gold PW. Thyroid function in bulimia nervosa. *Biol. Psychiatry*. 1993;33(6):408–414. https://doi.org/10.1016/0006-3223(93)90168-d

6. Strumia R. Eating disorders and the skin. *Clin Dermatol. 2013;31(1):*80-85. https://doi.org/10.1016/j.clindermatol.2011.11.011

7. Allison KC, Spaeth A, Hopkins CM. Sleep and Eating Disorders. *Curr. Psychiatry Rep.* 2016;*18*(10):92. https://doi.org/10.1007/s11920-016-0728-8

8. Anderson CB, Carter FA, McIntosh VV, Joyce PR, Bulik CM. Self-harm and suicide attempts in individuals with bulimia nervosa. *Eat Disord*. 2002;10(3):227-243. https://doi.org/10.1002/erv.472

9. de Vos JA, Radstaak M, Bohlmeijer ET, Westerhof GJ. Having an Eating Disorder and Still Being Able to Flourish? Examination of Pathological Symptoms and Well-Being as Two Continua of Mental Health in a Clinical Sample. *Front Psychol*. 2018;9:2145. https://doi.org/10.3389/fpsyg.2018.02145

10. Garcia SC, Mikhail ME, Keel PK, et al. Increased rates of eating disorders and their symptoms in women with major depressive disorder and anxiety disorders. *Int J. Eat Disord.* 2020;53(11):1844–1854. https://doi.org/10.1002/eat.23366

11. Sagiv E, Gvion Y. A multi factorial model of self-harm behaviors in Anorexia-nervosa and Bulimia-nervosa. *Compr Psychiatry.* 2020;96:152142. https://doi.org/10.1016/j.comppsych.2019.152142

12. Ulfvebrand S, Birgegård A, Norring C, Högdahl L, von Hausswolff-Juhlin Y. Psychiatric comorbidity in women and men with eating disorders results from a large clinical database. *Psychiatry Res*. 2015;230(2):294-299. https://doi.org/10.1016/j.psychres.2015.09.008

13. Qian J, Wu Y, Liu F, et al. An update on the prevalence of eating disorders in the general population: a systematic review and meta-analysis. *Eat Weight Disord*. 2022;27(2):415-428. https://doi.org/10.1007/s40519-021-01162-z

14. Galmiche M, Déchelotte P, Lambert G, Tavolacci MP. Prevalence of eating disorders over the 2000-2018 period: a systematic literature review. *Am J Clin Nutr*. 2019;109(5):1402-1413. https://doi.org/10.1093/ajcn/nqy342

15. Linardon J, Wade TD, de la Piedad Garcia X, Brennan L. The efficacy of cognitive-behavioral therapy for eating disorders: A systematic review and meta-analysis. *J Consult Clin Psychol*. 2017;85(11):1080-1094.

https://doi.org/10.1037/ccp0000245

16. Hamilton A, Mitchison D, Basten C, et al. Understanding treatment delay: Perceived barriers preventing treatment-seeking for eating disorders. *Aust N Z J Psychiatry*. 2022;56(3):248-259. https://doi.org/10.1177/00048674211020102

17. Cachelin FM, Striegel-Moore RH. Help seeking and barriers to treatment in a community sample of Mexican American and European American women with eating disorders. *Int J Eat Disord*. 2006;39(2):154-161. https://doi.org/10.1002/eat.20213

18. Vanheusden K, Mulder CL, van der Ende J, van Lenthe FJ, Mackenbach JP, Verhulst FC. Young adults face major barriers to seeking help from mental health services. *Patient Educ Couns*. 2008;73(1):97-104. https://doi.org/10.1016/j.pec.2008.05.006

19. Hart LM, Granillo MT, Jorm AF, Paxton SJ. Unmet need for treatment in the eating disorders: a systematic review of eating disorder specific treatment seeking among community cases. *Clin Psychol Rev*. 2011;31(5):727-735. https://doi.org/10.1016/j.cpr.2011.03.004

20. Mond JM, Hay PJ, Rodgers B, Owen C. Health service utilization for eating disorders: findings from a community-based study. *Int J Eat Disord*. 2007;40(5):399-408. https://doi.org/10.1002/eat.20382

21. Noordenbos G, Oldenhave A, Muschter J, Terpstra N. Characteristics and treatment of patients with chronic eating disorders. *Eat Disord*. 2002;10(1):15-29. https://doi.org/10.1080/106402602753573531

22. Oakley Browne MA, Wells JE, McGee MA; New Zealand Mental Health Survey Research Team. Twelve-month and lifetime health service use in Te Rau Hinengaro: The New Zealand Mental Health

Survey. *Aust N Z J Psychiatry*. 2006;40(10):855-864. https://doi.org/10.1080/j.1440-1614.2006.01904.x

23. Andersson G, Cuijpers P, Carlbring P, Riper H, Hedman E. Guided Internet-based vs. face-to-face cognitive behavior therapy for psychiatric and somatic disorders: a systematic review and meta-analysis. *World Psychiatry*. 2014;13(3):288-295. https://doi.org/10.1002/wps.20151

24. Hamatani S, Matsumoto K, Takahashi J, et al. Feasibility of guided internet-based cognitive behavioral therapy for patients with anorexia nervosa. *Internet Interv*. 2022;27:100504. https://doi.org/10.1016/j.invent.2022.100504

25. Fairburn CG, Cooper Z, Shafran R. Cognitive behaviour therapy for eating disorders: a "transdiagnostic" theory and treatment. *Behav Res Ther*. 2003;41(5):509-528. https://doi.org/10.1016/s0005-7967(02)00088-8

26. Fairburn CG. Cognitive behavior therapy and eating disorders. New York: Guilford Press; 2008. https://doi.org/10.1017/S1352465809990336

27. Boutelle KN, Monreal T, Strong DR, Amir N. An open trial evaluating an attention bias modification program for overweight adults who binge eat. *J Behav Ther Exp Psychiatry.* 2016;52:138-146. https://doi.org/10.1016/j.jbtep.2016.04.005

28. Godfrey KM, Gallo LC, Afari N. Mindfulness-based interventions for binge eating: a systematic review and meta-analysis. *J Behav Med*. 2015;38(2):348-362. https://doi.org/10.1007/s10865-014-9610-5

29. Mitchell KS, Mazzeo SE, Schlesinger MR, Brewerton TD, Smith BN. Comorbidity of partial and subthreshold ptsd among men and women with eating disorders in the national comorbidity survey-

replication study. *Int J Eat Disord*. 2012;45(3):307-315. https://doi.org/10.1002/eat.20965

30. McIntosh VV, Carter FA, Bulik CM, Frampton CM, Joyce PR. Five-year outcome of cognitive behavioral therapy and exposure with response prevention for bulimia nervosa. *Psychol Med*. 2011;41(5):1061-1071. https://doi.org/10.1017/S0033291710001583

31. Levinson CA, Zerwas S, Calebs B, et al. The core symptoms of bulimia nervosa, anxiety, and depression: A network analysis. *J Abnorm Psychol*. 2017;126(3):340-354. https://doi.org/10.1037/abn0000254

32. Hamatani S, Matsumoto K. A manual of cognitive behavioral therapy of bulimia nervosa for therapists. HOPE Project:2022. Available from: https://www.hopeproject.site/ [accessed 29-Jan-25]

33. Hamatani S, Matsumoto K, Ishibashi T, et al. Development of a culturally adaptable internet-based cognitive behavioral therapy for Japanese women with bulimia nervosa. *Front Psychiatry*. 2022;13:942936. https://doi.org/10.3389/fpsyt.2022.942936

34. Vlaescu G, Alasjö A, Miloff A, Carlbring P, Andersson G. Features and functionality of the Iterapi platform for internet-based psychological treatment. *Internet Interv*. 2016;6:107-114. https://doi.org/10.1016/j.invent.2016.09.006

35. Kessler RC, Berglund PA, Chiu WT, et al. The prevalence and correlates of binge eating disorder in the World Health Organization World Mental Health Surveys. *Biol Psychiatry*. 2013;73(9):904-914. https://doi.org/10.1016/j.biopsych.2012.11.020

36. Hamatani S, Tomotake M, Takeda T, et al. Impaired social cognition in anorexia nervosa patients. *Neuropsychiatr Dis Treat*. 2016;12:2527-2531. https://doi.org/10.2147/NDT.S116521

37. Hamatani S, Tomotake M, Takeda T, et al. Influence of cognitive function on quality of life in anorexia nervosa patients. *Psychiatry clin. neurosci.* 2017;71(5):328–335. https://doi.org/10.1111/pcn.12491

38. Jelinek L, Faissner M, Moritz S, Kriston L. Long-term efficacy of Metacognitive Training for Depression (D-MCT): A randomized controlled trial. *Br J Clin Psychol*. 2019;58(3):245-259. https://doi.org/10.1111/bjc.12213

39. Davis CA, Levitan RD, Reid C, et al. Dopamine for "wanting" and opioids for "liking": a comparison of obese adults with and without binge eating. *Obesity (Silver Spring)*. 2009;17(6):1220-1225. https://doi.org/10.1038/oby.2009.52

40. Nijs IM, Franken IH. Attentional Processing of Food Cues in Overweight and Obese Individuals. *Curr. Obes. Rep*. 2012;1(2):106–113. https://doi.org/10.1007/s13679-012-0011-1

41. MacLeod C, Clarke PJF. The attentional bias modification approach to anxiety intervention. *Clin Psychol Sci.* 2015;3(1):58-78. https://doi.org/10.1177/2167702614560749

42. Kuckertz JM, Amir N. Attention bias modification for anxiety and phobias: current status and future directions. *Curr psychiatry Rep.* 2015;*17*(2):9. https://doi.org/10.1007/s11920-014-0545-x

43. Anitha L, Abdulaziz Alhussaini A, Ibrahim Alsuwedan H, Faleh Alnefaie H, Abduallah Almubrek R, Abdulaziz Aldaweesh S. Bulimia Nervosa and Body Dissatisfaction in Terms of Self-Perception of Body Image [Internet]. Anorexia and Bulimia Nervosa. *IntechOpen*; 2019. Available from: http://dx.doi.org/10.5772/intechopen.84948. [accessed 29-Jan-25]

44. Waller G, Mountford VA. Weighing patients within cognitive-behavioural therapy for eating disorders:

How, when and why. *Behav Res and Ther.* 2015;*70*:1–10. https://doi.org/10.1016/j.brat.2015.04.004

45. Barakat S, Maguire S, Surgenor L, et al. The Role of Regular Eating and Self-Monitoring in the Treatment of Bulimia Nervosa: A Pilot Study of an Online Guided Self-Help CBT Program. *Behav Sci (Basel)*. 2017;7(3):39. https://doi.org/10.3390/bs7030039

46. Garner DM, Desai JJ, Desmond M, Wohlers J. Nutritional rehabilitation for eating disorders: river centre clinic program description. *Annal Nutr Disord Ther.* 2017;4(2): 1044. https://www.eat-26.com/wp-content/uploads/2022/08/2017-Garner-et-al-Nutr-Rehab-ED-RCC.pdf

47. Cooper PJ, Steere J. A comparison of two psychological treatments for bulimia nervosa: implications for models of maintenance. *Behav Res Ther*. 1995;33(8):875-885. https://doi.org/10.1016/0005-7967(95)00033-t

48. Rosen JC, Leitenberg H. Bulimia nervosa: treatment with exposure and response prevention. *Behav Ther.* 1982;13(1):117-124. https://psycnet.apa.org/doi/10.1016/S0005-7894(82)80055-5

49. Scott N, Hanstock TL, Thornton C. Dysfunctional self-talk associated with eating disorder severity and symptomatology. *J Eat Disord*. 2014;2:14. Published 2014 May 27. https://doi.org/10.1186/2050-2974-2-14

50. Higbed L, Fox JR. Illness perceptions in anorexia nervosa: a qualitative investigation. *Br J Clin Psychol*. 2010;49(Pt 3):307-325. https://doi.org/10.1348/014466509X454598

51. Hicks White AA, Pratt KJ, Cottrill C. The relationship between trauma and weight status among adolescents in eating disorder treatment. *Appetite*. 2018;129:62-69.

https://doi.org/10.1016/j.appet.2018.06.034

52. McIntosh VVW, Jordan J, Carter JD, et al. Psychotherapy for transdiagnostic binge eating: A randomized controlled trial of cognitive-behavioural therapy, appetite-focused cognitive-behavioural therapy, and schema therapy. *Psychiatry Res*. 2016;240:412-420. https://doi.org/10.1016/j.psychres.2016.04.080

53. Sheehan DV, Lecrubier Y, Sheehan KH, et al. The Mini-International Neuropsychiatric Interview (M.I.N.I.): the development and validation of a structured diagnostic psychiatric interview for DSM-IV and ICD-10. *J Clin Psychiatry*. 1998;59 Suppl 20:22-57.

54. Muramatsu K, Miyaoka H, Kamijima K, et al. The patient health questionnaire, Japanese version: validity according to the mini-international neuropsychiatric interview-plus. *Psychol Rep*. 2007;101(3 Pt 1):952-960. https://doi.org/10.2466/pr0.101.3.952-960

55. Fairburn CG, Beglin SJ. Assessment of eating disorders: interview or self-report questionnaire?. *Int J. Eat Dis.* 1994;*16*(4):363–370. https://doi.org/10.1002/1098-108X(199412)16:4<363::AID-EAT2260160405>3.0.CO;2-%23

56. Mitsui T, Yoshida T, Komaki G. Psychometric properties of the eating disorder examination-questionnaire in Japanese adolescents. *Biopsychosoc Med*. 2017;11:9. https://doi.org/10.1186/s13030-017-0094-8

57. Spitzer RL, Kroenke K, Williams JB. Validation and utility of a self-report version of PRIME-MD: the PHQ primary care study. Primary Care Evaluation of Mental Disorders. Patient Health Questionnaire. *JAMA*. 1999;282(18):1737-1744. https://doi.org/10.1001/jama.282.18.1737

58. Kroenke K, Spitzer RL, Williams JB. The PHQ-9: validity of a brief depression severity measure. *J Gen*

Intern Med. 2001;16(9):606-613. https://doi.org/10.1046/j.1525-1497.2001.016009606.x

59. Muramatsu K. Patient Health Questionnaire (PHQ-9, PHQ-15). Japanese version and generalized anxiety disorder-7 up to date. *Stud Clin Psychol.* 2014;7:35-39. https://core.ac.uk/reader/70372800

60. Spitzer RL, Kroenke K, Williams JB, Löwe B. A brief measure for assessing generalized anxiety disorder: the GAD-7. *Arch Intern Med*. 2006;166(10):1092-1097. https://doi.org/10.1001/archinte.166.10.1092

61. EuroQol Group. EuroQol--a new facility for the measurement of health-related quality of life. *Health Policy*. 1990;16(3):199-208. https://doi.org/10.1016/0168-8510(90)90421-9

62. Ikeda S, Shiroiwa T, Igarashi A, Noto S, Fukuda T, Aito S, Shimozuma K. Developing a Japanese version of the EQ-5D-5L value set. *J. Nat. Inst. Public Health.* 2015;64:47-55. https://www.niph.go.jp/journal/data/64-1/201564010008.pdf.

63. Lindner P, Frykheden O, Forsström D, et al. The Brunnsviken Brief Quality of Life Scale (BBQ): Development and Psychometric Evaluation. *Cogn. Behav. Ther.* 2016;45(3):182–195. https://doi.org/10.1080/16506073.2016.1143526

64. Hamatani S, Matsumoto K, Lindner P, Shimizu E, Mizuno Y, Andersson G. Reliability and validity of a Japanese version of the Brunnsviken Brief Quality of Life Scale. *PCN Rep*. 2024;3(1):e170. https://doi.org/10.1002/pcn5.170

65. Attkisson CC, Zwick R. The client satisfaction questionnaire. Psychometric properties and correlations with service utilization and psychotherapy outcome. *Eval Program Plann*. 1982;5(3):233-237. https://doi.org/10.1016/0149-7189(82)90074-x

66. Tachimori H, Ito H. Nihonngobann client satisfaction questionnaire 8 koumokubann no shinnraisei oyobi datousei no kenntou [Reliability and validity of the Japanese version of client satisfaction questionnaire]. *Seishin Igaku.* 1999;41(7):711-717. https://doi.org/10.11477/mf.1405905056

67. Hatcher RL, Gillaspy JA. Development and validation of a revised short version of the Working Alliance Inventory. *Psychother Res.* 2006;16(1):12–25. https://doi.org/10.1080/10503300500352500

68. Kawamura A, Irie T, Takebayashi Y, et al. A Japanese Version of Working Alliance Inventory-Short Revised (J-WAI-SR): Development and Examination of Psychometric Properties. 2020;46:191-202. https://doi.org/10.24468/jjbct.20-002

69. R Core Team. *R: A Language and Environment for Statistical Computing. R Foundation for Statistical Computing, Vienna, Austria: 2023.* Available from: https://www.r-project.org/ [accessed 29-Jan-25]

70. Schulz KF, Altman DG, Moher D; CONSORT Group. CONSORT 2010 statement: updated guidelines for reporting parallel group randomized trials. *Ann Intern Med*. 2010;152(11):726-732. https://doi.org/10.7326/0003-4819-152-11-201006010-00232

71. Pruessner L, Timm C, Barnow S, Rubel JA, Lalk C, Hartmann S. Effectiveness of a web-based cognitive behavioral self-help intervention for binge eating disorder: a randomized clinical trial. *JAMA Netw Open*. 2024;7(5):e2411127. https://doi.org/10.1001/jamanetworkopen.2024.11127

72. Hartmann S, Timm C, Barnow S, Rubel JA, Lalk C, Pruessner L. Web-based cognitive behavioral treatment for bulimia nervosa: a randomized clinical trial. *JAMA Netw Open*. 2024;7(7):e2419019. https://doi.org/10.1001/jamanetworkopen.2024.19019

73. Meule A, Hilbert A, de Zwaan M, Brähler E, Koch S, Voderholzer U. Cutoff scores of the Eating Disorder Examination-Questionnaire for the German population. *Int J Eat Disord*. 2024;57(3):602-610. https://doi.org/10.1002/eat.24133

74. Velkoff EA, Brown TA, Kaye WH, Wierenga CE. Using clinical cutoff scores on the eating disorder examination-questionnaire to evaluate eating disorder symptoms during and after naturalistic intensive treatment. *Eat Disord*. 2023;31(5):464-478. https://doi.org/10.1080/10640266.2023.2191488

75. Bang H, NiL, Davis CE. Assessment of blinding in clinical trials. *CCT.* 2004;*25*(2):143–156. https://doi.org/10.1016/j.cct.2003.10.016.

76. James KE, Bloch DA, Lee KK, Kraemer HC, Fuller RK. An index for assessing blindness in a multi-centre clinical trial: disulfiram for alcohol cessation--a VA cooperative study. *Stat Med*. 1996;15(13):1421–1434. https://doi.org/10.1002/(SICI)1097-0258(19960715)15:13<1421::AID-SIM266>3.0.CO;2-H

77. Faul F, Erdfelder E, Buchner A. et al. Statistical power analyses using G*Power 3.1: Tests for correlation and regression analyses. *Behav Res Methods*. 2009;41:1149–1160 (2009). https://doi.org/10.3758/BRM.41.4.1149

78. Ruwaard J, Lange A, Broeksteeg J, Renteria-Agirre A, Schrieken B, Dolan CV, Emmelkamp P. Online cognitive-behavioural treatment of bulimic symptoms: a randomized controlled trial. *Clin Psychol Psychother.* 2013;20(4):308-18. https://doi.org/10.1002/cpp.1767

79. Melisse B, Berg EVD, Jonge M, et al. Efficacy of Web-Based, Guided Self-help Cognitive Behavioral Therapy-Enhanced for Binge Eating Disorder: Randomized Controlled Trial. *J Med Internet Res*.

2023;25:e40472. https://doi.org/10.2196/40472

80. Loucas CE, Fairburn CG, Whittington C, Pennant ME, Stockton S, Kendall T. E-therapy in the treatment and prevention of eating disorders: A systematic review and meta-analysis. *Behav Res Ther*. 2014;63:122-131.https://doi.org/10.1016/j.brat.2014.09.011.

81. Wiberg AC, Ghaderi A, Danielsson HB, et al. Internet-based cognitive behavior therapy for eating disorders - Development and feasibility evaluation. *Internet Interv*. 2022;30:100570. https://doi.org/10.1016/j.invent.2022.100570

82. Burns PB, Rohrich RJ, Chung KC. The levels of evidence and their role in evidence-based medicine. *Plast. Reconstr. Surg*. 2011;128(1):305–310. https://doi.org/10.1097/PRS.0b013e318219c171

83. Strandskov SW, Ghaderi A, Andersson H, et al. Effects of Tailored and ACT-Influenced Internet-Based CBT for Eating Disorders and the Relation Between Knowledge Acquisition and Outcome: A Randomized Controlled Trial. *Behav Ther*. 2017;48(5):624-637. https://doi.org/10.1016/j.beth.2017.02.002

84. Högdahl L, Birgegård A, Norring C, de Man Lapidoth J, Franko MA, Björck C. Internet-based cognitive behavioral therapy for bulimic eating disorders in a clinical setting: Results from a randomized trial with one-year follow-up. *Internet Interv*. 2022;31:100598. Published 2022 Dec 21. https://doi.org/10.1016/j.invent.2022.100598

85. Takahashi F, Takegawa S, Okumura Y, Suzuki S. Actual condition survey on the implementation of cognitive behavioral therapy at psychiatric clinics in Japan. 2018. Available from: http://www.ftakalab.jp/wordpress/wp-

content/uploads/2011/08/japancbtclinic_report.pdf [accessed 29-Jun-25]
