# Supplementary material for: Guided Internet-Based Cognitive Behavior Therapy for Women With Bulimia Nervosa: A Randomized Clinical Trial
Source: JAMA Netw Open. 2025 Aug 5;8(8):e2525165. doi: 10.1001/jamanetworkopen.2025.25165 (PMC12326282; doi:10.1001/jamanetworkopen.2025.25165)
Supplement: Supplement 2. — eMethods eFigure. User Interface of the Bulimia Nervosa Internet-Based Cognitive Behavior Therapy Program eTable 1. Comparison of Baseline Characteristics Between Groups eTable 2. Baseline Outcome Comparison in Bulimia Nervosa Treatment: Intervention vs Control Groups eTable 3. Secondary Outcomes by Study Group Over Time eTable 4. Remission Rates and Odds Ratios for Each Group Based on the 2 Criteria eTable 5. Posttreatment Outcomes: Therapeutic Alliance and Client Satisfaction eTable 6. Treatment Adherence in the Intervention Group eTable 7. Clinical Variables by Study Group Over Time With Imputed Missing Values eTable 8. Comparison of Clinical Variables Using Analysis of Covariance eTable 9. Group Comparisons by Analysis of Covariance After Missing Data Imputation eTable 10. Per-Protocol Set (PPS) Analysis: Outcomes by Study Group Over Time eResults eTable 11. Results of Blinding Assessment eReferences [file jamanetwopen-e2525165-s002.pdf]

Supplemental Online Content

Hamatani S, Matsumoto K, Andersson G, et al. Guided internet-based cognitive behavior therapy for women with bulimia nervosa: a randomized clinical trial. *JAMA Netw Open*. 2025;8(8):e2525165. doi:10.1001/jamanetworkopen.2025.25165

eMethods
eFigure. User Interface of the Bulimia Nervosa Internet-Based Cognitive Behavior Therapy Program
eMethods
eTable 1. Comparison of Baseline Characteristics Between Groups
eTable 2. Baseline Outcome Comparison in Bulimia Nervosa Treatment: Intervention vs Control Groups
eTable 3. Secondary Outcomes by Study Group Over Time
eTable 4. Remission Rates and Odds Ratios for Each Group Based on the 2 Criteria
eTable 5. Posttreatment Outcomes: Therapeutic Alliance and Client Satisfaction
eTable 6. Treatment Adherence in the Intervention Group
eTable 7. Clinical Variables by Study Group Over Time With Imputed Missing Values
eTable 8. Comparison of Clinical Variables Using Analysis of Covariance
eTable 9. Group Comparisons by Analysis of Covariance After Missing Data Imputation
eResults
eTable 10. Per-Protocol Set (PPS) Analysis: Outcomes by Study Group Over Time
eTable 11. Results of Blinding Assessment
eReferences

This supplemental material has been provided by the authors to give readers additional information about their work.

© 2025 Hamatani S et al. *JAMA Network Open*.

eMethods

Justification for Specific Inclusion and Exclusion Criteria

Inclusion of only female participants

Bulimia nervosa is a condition that predominantly affects women. Although exceptions exist, the typical clinical presentation of bulimia nervosa is more commonly observed in women. The ICBT program used in this study was developed specifically for women with typical BN, and all photographs and illustrations in the program feature female figures (see eFigure). Limiting participation to women was intended to promote sample homogeneity and enhance the generalizability of the results to the primary clinical population.

Exclusion of individuals with antisocial personality disorder

This exclusion criterion was included based on the concern that individuals with antisocial traits may have difficulty providing reliable self-reports and complying with study procedures, including weekly access, review of intervention materials, completion of homework assignments, and outcome assessments.

Exclusion of individuals unwilling to engage in exposure

Exposure to the objects of one's anxiety or fear is included in the current intervention. Individuals who refuse to engage in this component are essentially not consenting to the treatment content. Accordingly, those who cannot agree to the inclusion of exposure were considered ineligible for the study.

Eligibility Assessment Process

Participants had been diagnosed by clinicians at the research sites (university hospitals) and were introduced to this research project while continuing their usual treatment. For in-person eligibility assessments, the first author (SH) conducted assessments at Fukui University, the second author (KM) at Kagoshima University, and consortium authors (RK, TY, and KM) at Chiba University and Tokushima University. The eligibility assessors were clinical psychology experts, with four holding PhDs and one holding an MA. Women wishing to participate in this study via the online form were required to mail a referral letter from their primary psychiatrist to the study secretariat. Information regarding their eligibility was collected via telephone or video conference by members of the study secretariat, led by the first author (SH), and the final eligibility determination based on this information was made through consensus among the first author (SH), the second author (KM), and the senior author (YM) who is a medical doctor and a specialist in psychiatry, holding a PhD. In cases where eligibility was difficult to determine, the primary physician was contacted directly, and eligibility was decided through discussion via video conference; two participants were enrolled through this process.

© 2025 Hamatani S et al. *JAMA Network Open*.

Changes from the Original Protocol

The protocol originally specified analysis of covariance (ANCOVA) as the primary analysis method, as outlined in the published protocol paper.[1] However, to better align with the evolving understanding of data characteristics and enhance analytical precision, we updated the protocol and adopted linear mixed models (LMM) as the primary analysis method, following recommendations from prior studies.[2,3] Analysis of covariance (ANCOVA) was used for sensitivity analysis.

© 2025 Hamatani S et al. *JAMA Network Open*.

eFigure. User Interface of the Bulimia Nervosa Internet-Based Cognitive Behavior Therapy Program

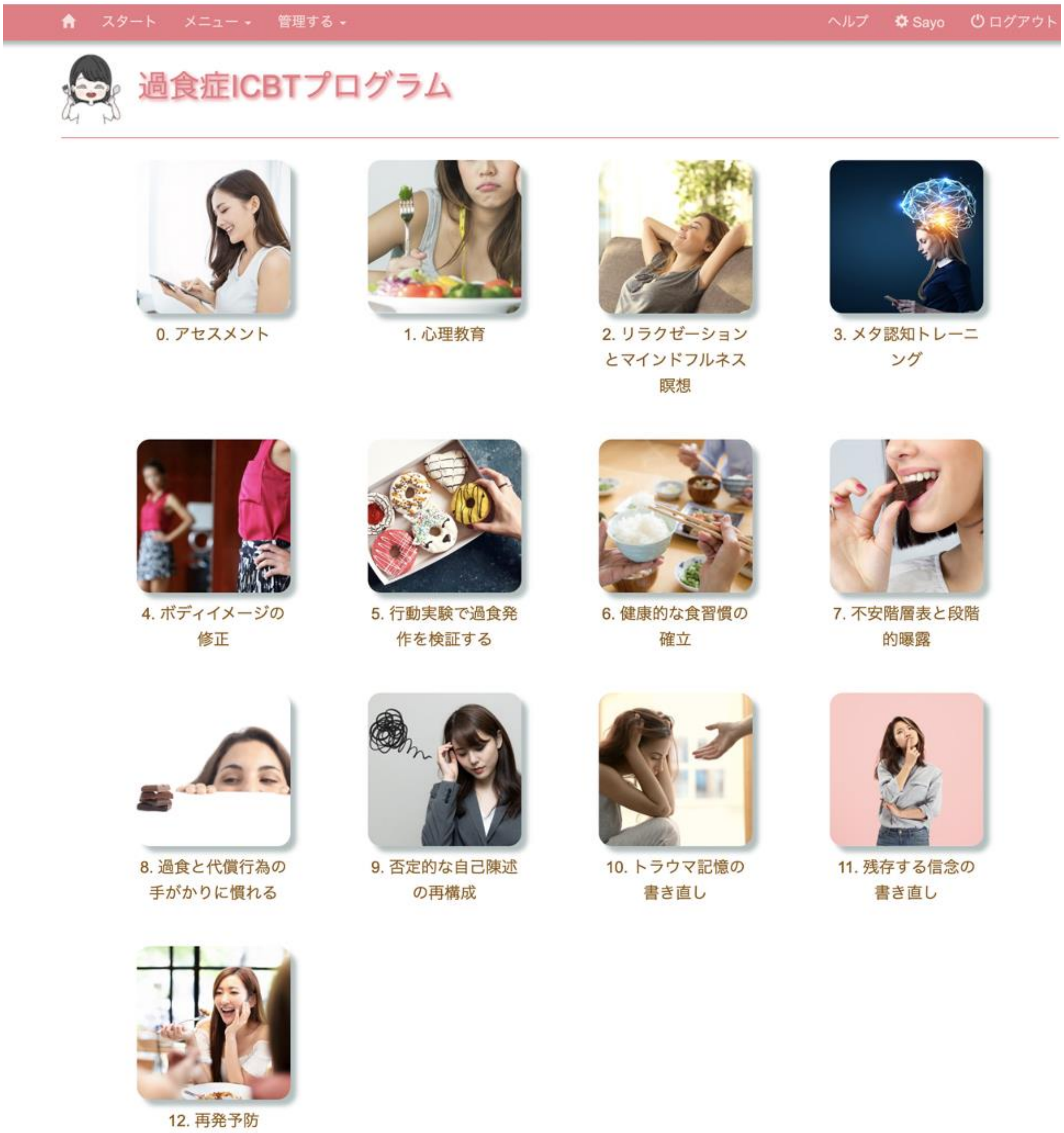

Note: In addition to the structured modules shown above, therapist guidance was provided via asynchronous messaging. The guidance primarily consisted of supportive encouragement, clarification on how to complete homework assignments, responses to participants' questions, and brief instructions regarding upcoming sessions.

© 2025 Hamatani S et al. *JAMA Network Open*.

eMethods

Assessment Procedure and Rationale for One-Week Outcome Measurement

Assessment Procedure and Rationale

The primary outcome in this study was the weekly combined frequency of binge eating and compensatory behaviors, assessed via structured telephone interviews conducted by independent raters. This approach was chosen to minimize potential bias associated with self-reported outcomes. Interview appointments were scheduled in advance via email, and if a participant was unavailable at the scheduled time, a voicemail was left and the interview was rescheduled. Telephone interviews were employed to ensure a standardized assessment format while accommodating the remote nature of the intervention, thereby supporting both feasibility and data quality. This procedure contributed to a high post-assessment completion rate at 12 weeks, with data obtained from all participants except two who withdrew before starting the intervention, resulting in only a minimal amount of missing data.

Justification for the weekly combined frequency of binge eating and compensatory behaviors

The rationale for using the weekly combined frequency of binge eating and compensatory behaviors as the primary outcome was to improve the accuracy of symptom reporting. In face-to-face CBT for bulimia nervosa (BN), patients are typically asked to keep food diaries and record episodes of binge eating and compensatory behaviors. However, based on our clinical experience, many patients complete these records retrospectively just before their sessions, relying on memory rather than real-time tracking. Therefore, a shorter recall period of one week was adopted to allow for more accurate and reliable measurement of symptom frequency.

Assessment of Blinding

The success of blinding was evaluated using the methods of both James[4] and Bang.[5] Independent assessors were asked at the final data collection point, “What type of treatment do you think the participant received?” Possible responses included “guided ICBT,” “UC,” or “I do not know.”

The Bang blinding index (BI), ranging from −1 to 1, was employed to assess the success of blinding. [5] A BI score of 0 indicates perfect blinding, 1 represents complete unblinding, and −1 suggests that all assessors guessed their treatment allocation incorrectly. If the one-sided confidence interval for the Bang index excluded 0, the study was considered to have inadequate blinding. In addition, the James blinding index was calculated to provide further insight into blinding success. The James index is a continuous measure ranging from 0 to 1, where a score of 0.5 indicates perfect blinding. Higher values suggest a greater deviation from blinding. Confidence intervals for both indices were calculated to interpret the adequacy of blinding in this study. The free statistical software R, using the 'BI' package, was utilized for these analyses.[6]

© 2025 Hamatani S et al. *JAMA Network Open*.

eTable 1. Comparison of Baseline Characteristics Between Groups

Characteristic	Participants, no. (%)	Intervention group（N = 31）	Control group (N = 30)	Test statistic	*P* value
Age, mean (SD), y	27.79 (9.0)	28.32 (10.28)	27.23 (7.66)	0.468	.64
Body mass index, mean (SD)	21.10 (3.6)	20.73 (3.9)	21.49 (3.3)	-0.813	.42
Number of years of education, mean (SD), y	13.84 (2.78)	13.73 (2.55)	13.95 (3.0)	-0.301	.77
Higher educational level	61	31	30	1.351a	.85
Junior high school	8 (13.1)	4 (12.9)	4 (13.3)	-	-
High school	24 (39.3)	13 (41.9)	11 (36.7)	-	-
Junior college/technical school	6 (9.8)	2 (6.5)	4 (13.3)	-	-
University degree	20 (32.8)	11 (35.5)	9 (30.0)	-	-
Graduate school	3 (4.9)	1 (3.2)	2 (6.7)	-	-
Occupation status				0.349a	.84
Employed	31 (50.8)	15 (48.4)	16 (53.3)	-	-
Student	18 (29.5)	7 (22.6)	5 (16.7)	-	-
Unemployed	12 (19.7)	9 (29.0)	9 (30.0)	-	-
Age of onset, mean (SD), y	18.56 (4.93)	19.23 (5.86)	17.87 (3.70)	1.079	.29
Duration of illness, mean (SD), y	9.27 (8.82)	9.11 (9.23)	9.43 (8.52)	-0.138	.89

© 2025 Hamatani S et al. *JAMA Network Open*.

Characteristic	Participants, no. (%)	Intervention group （N = 31）	Control group (N = 30)	Test statistic	*P* value
Marital status					
Single	47 (77.1)	23 (74.2)	24 (80.0)	-	.76
Married	14 (23.0)	8 (25.8)	6 (20.0)		
Divorced (yes)	4 (6.6)	3 (9.7)	1 (3.3)	-	.61
Drinking alcohol (yes)	25 (41.0)	13 (41.9）	12 (40.0)	-	1.00
Smoking (yes)	5 (8.2)	1 (3.2)	4 (13.3)	-	.20
Family mental illness history (yes)	17 (27.9)	8 (25.8)	9 (30.0)	-	.78
Psychotropic medication	28 (45.9)	14 (45.2)	14 (46.7)	-	1.00
Comorbidities (yes)	28 (45.9)	14 (45.2)	14 (46.7)	-	.91
Depressive disorders	14 (23.0)	7 (22.6)	7 (23.3)	-	.94
Dysthymic disorder	4 (6.6)	2 (6.5)	2 (6.7)	-	1.00
Bipolar disorder	2 (3.3)	1 (3.2)	1 (3.3)	-	1.00
Panic disorder	1 (1.6)	0 (0.0)	1 (3.3)	-	.49
Agoraphobia	6 (9.8)	3 (9.7)	3 (10.0)	-	1.00
Social anxiety disorders	7 (11.5)	2 (6.5)	5 (16.7)	-	.26
Obsessive-compulsive disorder	1 (1.6)	1 (3.2)	0 (0.0)	-	1.00
Generalized anxiety disorder	6 (9.8)	4 (12.9)	2 (6.7)	-	.67
Note: Independent samples t-test and χ2-test or Fisher’s test were performed.					

© 2025 Hamatani S et al. *JAMA Network Open*.

eTable 2. Baseline Outcome Comparison in Bulimia Nervosa Treatment: Intervention vs Control Groups

	Mean (SD)			
Outcome, group	Intervention (n=31)	Control (n=30)	Test statistic	*P* value
Binge eating and compensatory behavior episodes	19.13 (16.61)	14.27 (11.61)	1.32	.19
Binge eating episodes	8.48 (7.38)	6.30 (5.96)	1.27	.21
Compensatory behavior episodes	10.77 (9.77)	7.97 (6.54)	1.31	.19
EDE-Q global score	3.61 (1.23)	3.95 (0.97)	-1.18	.24
PHQ-9	12.45 (6.04)	13.27 (6.37)	-0.51	.61
GAD-7	7.10 (5.53)	8.47 (5.25)	-0.99	.33
EQ-5D-5L	0.81 (0.14)	0.83 (0.10)	0.50	.62
BBQ	28.58 (19.53)	28.63 (22.39)	-0.10	.99

Abbreviations: BBQ, Brunnsviken Brief Quality of Life Scale; EDE-Q, Eating Disorder Examination Questionnaire; EQ-5D-5L, EuroQol 5 Dimensions 5 Levels; GAD-7, General Anxiety Disorder-7, PHQ-9: Patient Health Questionnaire-9.

© 2025 Hamatani S et al. *JAMA Network Open*.

eTable 3. Secondary Outcomes by Study Group Over Time

	Mixed model	Effect size	*P*-value
	Adjusted difference (95%CI)	Cohen's *d* (95%CI)	
**Secondary outcomes**			
PHQ-9	-0.42 (-3.11 to 2.27)	-0.08 (-0.59 to 0.43)	.75
GAD-7	-0.43 (-2.91 to 2.06)	-0.09 (-0.06 to 0.42)	.73
EQ-5D-5L	0.00 (-0.06 to 0.07)	0.03 (-0.48 to 0.54)	.90
BBQ	-6.11 (-13.97 to 1.75)	-0.34 (-0.86 to 0.17)	.13

Abbreviations: BBQ, Brunnsviken Brief Quality of Life Scale; EDE-Q, Eating Disorder Examination Questionnaire; EQ-5D-5L, EuroQol 5 Dimensions 5 Levels; GAD-7, General Anxiety Disorder-7; PHQ-9, Patient Health Questionnaire-9.

Note: Secondary outcome results were not adjusted for multiplicity and should be interpreted as exploratory.

© 2025 Hamatani S et al. *JAMA Network Open*.

eTable 4. Remission Rates and Odds Ratios for Each Group Based on the 2 Criteria

Criteria of remission	Group	In remission, n (%)	Not in remission, n (%)	Total	Odds ratio (95% CI)	*P*-value
The EDE-Q score ≤ 2.34						
	Intervention group	13 (44.8)	16 (55.2)	29	5.28 (1.47 to 19.03)	.008
	Control group	4 (13.3)	26 (86.7)	30		
The EDE-Q score ≤ 2.80						
	Intervention group	16 (55.2)	13 (44.8)	29	8.00 (2.22 to 28.83)	<.001
	Control group	4 (13.3)	26 (86.7)	30		

Abbreviations: EDE-Q, Eating Disorder Examination Questionnaire.

© 2025 Hamatani S et al. *JAMA Network Open*.

eTable 5. Posttreatment Outcomes: Therapeutic Alliance and Client Satisfaction

Outcome		Mean (SD)
		(n=29)
WAI-SF		55.66 (18.17)
CSQ		22.76 (6.41)
	1. How would you rate the quality of service you received?	72%
	2. Did you get the kind of service you wanted?	55%
	3. To what extent has our service met your needs?	59%
	4. If a friend were in need of similar help, would you recommend our service to him or her?	59%
	5. How satisfied are you with the amount of help you received?	66%
	6. Have the services you received helped you to deal more effectively with your problems?	59%
	7. In an overall, general sense, how satisfied are you with the service you received?	62%
	8. If you were to seek help again, would you come back to our service?	72%

Abbreviations: WAI-SF, Working Alliance Inventory - Short Form; CSQ-8, Client Satisfaction Questionnaire (8 items). Percentages represent the proportion of respondents who rated the item as “3” or “4” on a 4-point Likert scale, where “1” indicates the lowest satisfaction and “4” indicates the highest satisfaction.

© 2025 Hamatani S et al. *JAMA Network Open*.

eTable 6. Treatment Adherence in the Intervention Group

Module	Access Rate to the ICBT Content (%)	Worksheet Filled in (%)	Average Worksheet Completion Rate (%)
Module1	100.0	82.8	82.8
Module2	100.0	N/A	N/A
Module3	100.0	100.0	51.2
Module4	96.6	86.2	80.5
Module5	96.6	65.5	65.5
Module6	93.1	N/A	N/A
Module7	93.1	72.4	63.8
Module8	89.7	69.0	44.1
Module9	86.2	75.9	61.6
Module10	89.7	69.0	59.4
Module11	82.8	72.4	60.7
Module12	72.4	65.5	55.2
Mean	91.7	75.9	68.1

Abbreviations: ICBT, internet-based cognitive behavioral therapy.

Note: Worksheet Filled in rate (%) indicates the percentage of participants who entered information in at least one worksheet in each module. Average Worksheet Completion Rate (%) represents the mean percentage of worksheets completed by each participant in each module.

© 2025 Hamatani S et al. *JAMA Network Open*.

eTable 7. Clinical Variables by Study Group Over Time With Imputed Missing Values

	Mixed model	Effect size	
Outcome	Adjusted difference (95% CI)	Cohen's *d* (95% CI)	*P*-value
**Primary outcome**			
Binge eating and compensatory behavior episodes	9.75 (2.61 to 16.89)	0.74 (0.22 to 1.26)	.009
**Secondary outcomes**			
Binge eating episodes	3.32 (0.15 to 6.50)	0.53 (0.02 to 1.05)	.04
Compensatory behavior episodes	6.58 (1.49 to 11.67)	0.83 (0.31 to 1.35)	.01
EDE-Q global score	0.61 (0.15 to 1.07)	0.60 (0.08 to 1.11)	.01
PHQ-9	-0.64 (-3.30 to 2.03)	-0.11 (-0.62 to 0.39)	.63
GAD-7	-0.58 (-3.02 to 1.86)	-0.12 (-0.62 to 0.38)	.64
EQ-5D-5L	0.01 (-0.06 to 0.08)	0.08 (-0.42 to 0.58)	.74
BBQ	-6.25 (-14.20 to 1.70)	-0.35 (-0.85 to 0.16)	.12

Abbreviations: BBQ, Brunnsviken Brief Quality of Life Scale; EDE-Q, Eating Disorder Examination Questionnaire; EQ-5D-5L, EuroQol 5 Dimensions 5 Levels; GAD-7, General Anxiety Disorder-7; PHQ-9, Patient Health Questionnaire-9.

© 2025 Hamatani S et al. *JAMA Network Open*.

eTable 8. Comparison of Clinical Variables Using Analysis of Covariance

	Mean (SD)		Within-group effects	Interaction effects	ANCOVA	
Outcome, group	Pretreatment （n=61)	Posttreatment (n=59)	Cohen’s *d* (95% CI)	Cohen’s *d* (95% CI)	*F*-value	*P*-value
**Primary outcome**, Binge eating and compensatory behavior episodes						
Intervention group	19.13 (16.61)	10.66 (12.35)	-0.58 (-1.10 to -0.06)	-0.71 (-0.91 to -0.51)	10.78	.002
Control group	14.27 (11.61)	15.70 (14.17)	0.11 (-0.40 to 0.62)			
**Secondary outcomes**						
Binge eating episodes						
Intervention group	8.48 (7.38)	5.41 (5.36)	-0.48 (-0.99 to 0.04)	-0.51 (-0.70 to -0.32)	3.84	.06
Control group	6.30 (5.96)	6.57 (7.17)	0.04 (-0.47 to 0.55)			
Compensatory behavior episodes						
Intervention group	10.77 (9.77)	5.24 (7.26)	-0.64 (-1.16 to -0.12)	-0.83 (-1.04 to -0.62)	13.33	<.001
Control group	7.97 (6.54)	9.13 (8.13)	0.16 (-0.35 to 0.66)			
EDE-Q global score						
Intervention group	3.61 (1.23)	2.76 (1.38)	-0.65 (-1.17 to -0.13)	-0.54 (-0.74 to -0.35)	10.25	.002
Control group	3.95 (0.97)	3.75 (1.16)	-0.19 (-0.69 to 0.32)			

© 2025 Hamatani S et al. *JAMA Network Open*.

	Mean (SD)		Within-group effects	Interaction effects	ANCOVA	
Outcome, group	Pretreatment （n=61)	Posttreatment (n=59)	Cohen’s *d* (95% CI)	Cohen’s *d* (95% CI)	*F*-value	*P*-value
PHQ-9						
Intervention group	12.45 (6.04)	12.07 (6.60)	-0.06 (-0.57 to 0.45)	0.09 (-0.09 to 0.27)	0.00	.96
Control group	13.27 (6.37)	12.33 (5.86)	-0.15 (-0.66 to 0.35)			
GAD-7						
Intervention group	7.10 (5.53)	7.62 (4.67)	0.10 (-0.41 to 0.61)	0.11 (-0.07 to 0.29)	0.02	.88
Control group	8.47 (5.25)	8.43 (4.96)	-0.01 (-0.51 to 0.50)			
EQ-5D-5L						
Intervention group	0.81 (0.14)	0.81 (0.14)	0.00 (-0.51 to 0.51)	0.00 (-0.18 to 0.18)	0.11	.74
Control group	0.83 (0.10)	0.83 (0.14)	0.00 (-0.51 to 0.51)			
BBQ						
Intervention group	28.58 (19.53)	34.34 (22.93)	0.27 (-0.24 to 0.78)	0.29 (0.11 to 0.47)	2.55	.12
Control group	28.63 (22.39)	28.23 (20.73)	-0.02 (-0.52 to 0.49)			

Abbreviations: BBQ, Brunnsviken Brief Quality of Life Scale; EDE-Q, Eating Disorder Examination Questionnaire; EQ-5D-5L, EuroQol 5 Dimensions 5 Levels; GAD-7, General Anxiety Disorder-7; PHQ-9, Patient Health Questionnaire-9.

© 2025 Hamatani S et al. *JAMA Network Open*.

eTable 9. Group Comparisons by Analysis of Covariance After Missing Data Imputation

	Mean (SD)		Within-group effects	Interaction effects	ANCOVA	
Outcome, group	Pretreatment （n=61)	Posttreatment (n=61)	Cohen's *d* (95% CI)	Cohen's *d* (95% CI)	*F*-value	*P*-value
**Primary outcome,** Binge eating and compensatory behavior episodes						
Intervention group	19.13 (16.61)	10.81 (11.95)	-0.58 (-1.08 to -0.07)	-0.71 (0.51 to -0.51)	10.08	.002
Control group	14.27 (11.61)	15.70 (14.17)	0.11 (-0.40 to 0.62)			
**Secondary outcomes**						
Binge eating episodes						
Intervention group	8.48 (7.38)	5.43 (5.18)	-0.48 (-0.98 to 0.03)	-0.51 (-0.70 to -0.32)	2.98	.09
Control group	6.30 (5.96)	6.57 (7.17)	0.04 (-0.47 to 0.55)			
Compensatory behavior episodes						
Intervention group	10.77 (9.77)	5.36 (7.03)	-0.64 (-1.15 to -0.13)	-0.82 (-1.03 to -0.62)	13.62	<.001
Control group	7.97 (6.54)	9.13 (8.13)	0.16 (-0.35 to 0.66)			
EDE-Q global score						
Intervention group	3.61 (1.23)	2.81 (1.35)	-0.62 (-1.13 to -0.11)	-0.51 (-0.70 to -0.32)	8.16	.006
Control group	3.95 (0.97)	3.75 (1.16)	-0.19 (-0.69 to 0.32)			

© 2025 Hamatani S et al. *JAMA Network Open*.

	Mean (SD)		Within-group effects	Interaction effects	ANCOVA	
Outcome, group	Pretreatment （n=61)	Posttreatment (n=61)	Cohen’s *d* (95% CI)	Cohen’s *d* (95% CI)	*F*-value	*P*-value
PHQ-9						
Intervention group	12.45 (6.04)	12.15 (6.38)	-0.05 (-0.55 to 0.45)	0.10 (-0.07 to 0.28)	0.07	.79
Control group	13.27 (6.37)	12.33 (5.86)	-0.15 (-0.66 to 0.35)			
GAD-7						
Intervention group	7.10 (5.53)	7.64 (4.54)	0.11 (-0.39 to 0.61)	0.11 (-0.06 to 0.29)	0.00	.98
Control group	8.47 (5.25)	8.43 (4.96)	-0.01 (-0.51 to 0.50)			
EQ-5D-5L						
Intervention group	0.81 (0.14)	0.80 (0.14)	-0.07 (-0.57 to 0.43)	-0.02 (-0.20 to 0.16)	0.31	.58
Control group	0.83 (0.10)	0.83 (0.14)	0.00 (-0.51 to 0.51)			
BBQ						
Intervention group	28.58 (19.53)	34.43 (22.16)	0.28 (-0.22 to 0.78)	0.29 (0.11 to 0.48)	2.60	.11
Control group	28.63 (22.39)	28.23 (20.73)	-0.02 (-0.53 to 0.49)			

Abbreviations: BBQ, Brunnsviken Brief Quality of Life Scale; EDE-Q, Eating Disorder Examination Questionnaire; EQ-5D-5L, EuroQol 5 Dimensions 5 Levels; GAD-7, General Anxiety Disorder-7; PHQ-9, Patient Health Questionnaire-9.

© 2025 Hamatani S et al. *JAMA Network Open*.

eResults

Results of Per Protocol Set (PPS) Analysis

Participant Flow

A total of 31 participants were enrolled in the intervention group. Among them, two participants dropped out before the intervention, three did not complete at least 80% of the program modules, and three failed to meet the worksheet completion criteria. Consequently, eight participants were excluded, leaving 23 protocol-adherent participants for PPS analysis. In the control group, all 30 participants completed the trial and their data were included in the analysis.

eTable 10. Per-Protocol Set (PPS) Analysis: Outcomes by Study Group Over Time

	Mixed model	Effect size	*P*-value
Outcome	Adjusted difference (95%CI)	Cohen's *d* (95%CI)	
**Primary outcome,** Binge eating and compensatory behavior episodes	11.00 (3.27 to 18.73)	0.81 (0.24 to 1.37)	.007
**Secondary outcomes**			
Binge eating episodes	3.96 (0.55 to 7.37)	0.64 (0.08 to 1.19)	.02
Compensatory behavior episodes	7.21 (1.64 to 12.78)	0.88 (0.31 to 1.45)	.01
EDE-Q global scare	0.69 (0.18 to 1.20)	0.71 (0.15 to 1.27)	.01
PHQ-9	-0.15 (-3.12 to 2.81)	-0.27 (-0.57 to 0.52)	.92
GAD-7	-0.38 (-3.15 to 2.39)	-0.08 (-0.62 to 0.46)	.78
EQ-5D-5L	-0.02 (-0.09 to 0.05)	-0.20 (-0.74 to 0.35)	.52
BBQ	-7.49 (-15.94 to 0.97)	-0.43 (-0.98 to 0.12)	.08

Abbreviations: BBQ, Brunnsviken Brief Quality of Life Scale; EDE-Q, Eating Disorder Examination Questionnaire; EQ-5D-5L, EuroQol 5 Dimensions 5 Levels; GAD-7, General Anxiety Disorder-7; PHQ-9, Patient Health Questionnaire-9.

© 2025 Hamatani S et al. *JAMA Network Open*.

Results of Observed Power Analyses

The observed power for the primary outcome, which is the total score of binge eating and compensatory behaviors, was 0.896, and the observed power for the total EDE-Q score, measuring the severity of eating disorders, was 0.881. While the observed power for binge eating behavior was somewhat low at 0.485, the observed power for compensatory behavior was high at 0.948. These values are reported for reference only, and their interpretive value is limited.

Blinding Assessment Results

For the James BI, the estimated value (0.508) was very close to the success criterion for blinding (0.5), and the confidence interval included 0.5 (eTable 11). [4] Similarly, for the Bang BI, the estimated values for both groups generally fell within the success range for blinding (-0.2 to 0.2), and the confidence intervals included 0.[5] Based on these results, there was no evidence suggesting that blinding was compromised (eTable 11).

eTable 11. Results of Blinding Assessment

	Estimate	Std. Error	95% Confidence Interval
James Blinding Index	0.508	0.064	0.383, 0.634
Bang Blinding Index Treatment	0.207	0.141	-0.070, 0.484
Bang Blinding Index Control	0.200	0.173	-0.138, 0.538

© 2025 Hamatani S et al. *JAMA Network Open*.

eReferences

1. Hamatani S, Matsumoto K, Andersson G, et al. Guided internet-based cognitive behavioral therapy for women with bulimia nervosa: protocol for a multicenter randomized controlled trial. *JMIR Res Protoc*. 2023;12:e49828. DOI: 10.2196/49828
2. Pruessner L, Timm C, Barnow S, Rubel JA, Lalk C, Hartmann S. Effectiveness of a web-based cognitive behavioral self-help intervention for binge eating disorder: a randomized clinical trial. *JAMA Netw Open.* 2024;7(5):e2411127. doi: 10.1001/jamanetworkopen.2024.11127
3. Hartmann S, Timm C, Barnow S, Rubel JA, Lalk C, Pruessner L. Web-based cognitive behavioral treatment for bulimia nervosa: a randomized clinical trial. *JAMA Netw Open.* 2024;7(7):e2419019. DOI: 10.1001/jamanetworkopen.2024.19019
4. James KE, Bloch DA, Lee KK, Kraemer HC, Fuller RK. An index for assessing blindness in a multi-centre clinical trial: disulfiram for alcohol cessation--a VA cooperative study. *Stat Med*. 1996;15(13):1421-1434. DOI: 10.1002/(SICI)1097-0258(19960715)15:13<1421::AID-SIM266>3.0.CO;2-H
5. Bang H, Ni L, Davis CE. Assessment of blinding in clinical trials. *Control Clin Trials*. 2004;25(2):143-156. https://doi.org/10.1016/j.cct.2003.10.016
6. Schwartz M, Mercaldo N. BI: Blinding Assessment Indexes for Randomized, Controlled, Trials. Version 1.2.0. CRAN. 2022-12-4. doi: 10.32614/CRAN.package.BI. Available from: https://github.com/marcschwartz/BI. [accessed 5-May-25]

© 2025 Hamatani S et al. *JAMA Network Open*.
