# Supplementary material for: Guided Internet-Based Cognitive Behavior Therapy for Women With Bulimia Nervosa: A Randomized Clinical Trial
Source: JAMA Netw Open. 2025 Aug 5;8(8):e2525165. doi: 10.1001/jamanetworkopen.2025.25165 (PMC12326282; doi:10.1001/jamanetworkopen.2025.25165)
Supplement: Supplement 3. — Data Sharing Statement [file jamanetwopen-e2525165-s003.pdf]

Data Sharing Statement

Hamatani. Guided Internet-Based Cognitive Behavior Therapy for Women With Bulimia Nervosa. *JAMA Netw Open*. Published August 05, 2025.
doi:10.1001/jamanetworkopen.2025.25165

Data
Additional Information: Trial Registration：UMIN00048732 (https://center6.umin.ac.jp/cgi-open-bin/ctr/ctr_view.cgi?recptno=R000055522).
Data available: Yes
Data types: Deidentified participant data
How to access data: sayoh@u-fukui.ac.jp
When available: With publication

Supporting Documents
Document types: None

Additional Information
Who can access the data: Data will be made available to researchers whose proposed use of the data has been approved.
Types of analyses: Data will be made available for analyses for a specified purpose.
Mechanisms of data availability: Data will be made available both after approval of a proposal and with a signed data access agreement.
